# Asymmetric catalysis in direct nitromethane-free Henry reactions

**DOI:** 10.1039/c9ra10263a

**Published:** 2020-01-13

**Authors:** Lin Dong, Fen-Er Chen

**Affiliations:** Research Center for Drug Precision Industrial Technology, West China School of Pharmacy, Sichuan University Chengdu 610041 P. R. of China dongl@scu.edu.cn; Engineering Center of Catalysis and Synthesis for Chiral Molecules, Department of Chemistry, Fudan University Shanghai 200433 P. R. of China rfchen@fudan.edu.cn

## Abstract

A great number of reports have described asymmetric catalytic Henry reactions using nitromethanes as pronucleophiles, but far more challenging is diastereoselective catalytic Henry reactions using substituted higher nitroalkanes instead of nitromethane to generate chiral β-nitro alcohol scaffolds with four adjacent stereogenic centers in a one-pot operation. This review summarizes the current state and applications of such reactions involving complex nitroalkane coupling with various carbonyl compounds for resolving double chiral centers with high enantio- and diastereoselectivities.

## Abstract 2

This review summarizes the current state and applications of catalytic Henry reactions involving complex nitroalkanes coupling with various carbonyl compounds to generate chiral β-nitro alcohol scaffolds with four adjacent stereogenic centers.

## Graphical abstract

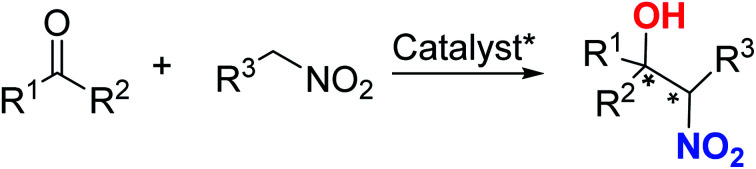

## 1. Introduction

Over the past two decades, asymmetric catalytic Henry reactions have been established as an integral part of asymmetric catalysis.^1,2^ During this period, a lot of reviews using nitromethanes as pronucleophiles have been reported. Despite the significant developments of the high enantioselectivity of these single chiral center products, many synthetic challenges arising from the demand for complex nitroalkanes instead of simply using nitromethanes as pronucleophiles have inspired chemists to construct chiral β-nitro alcohol scaffolds with four adjacent stereogenic centers in a one-pot operation. In particular, applications of these nitromethane-free reactions in the synthesis of chiral β-nitro alcohols have created new possibilities for the asymmetric preparation of natural products, pharmaceutical drugs, and bioactive molecules in academic and industrial settings.^3^

Since the seminal work reported by Shibasaki using metal/chiral ligand complexes in catalytic asymmetric nitroaldol reactions in 1992,^4^ chemists have overcome myriad synthetic challenges by developing various efficient catalytic systems, particularly chiral metal catalysts;^1,2,5^ chiral ligands such as Schiff bases, tetrahydrosalens, amino alcohols, and diamines;^1^ and small organic molecules such as guanidine, cinchona alkaloid-derived organocatalysts and quaternary ammonium salts.^2^ 2D materials are also a powerful platform to construct efficient catalysts for various reactions, such as asymmetric catalysis, CO_2_ reduction, CO oxidation.^6^

The present review aims to describe the impressive growth in this rapidly expanding field. It examines the current state of direct asymmetric catalytic Henry reactions involving higher nitroalkanes rather than simple nitromethanes coupling with various carbonyl compounds that control the stereochemistry at double chiral centers. The review will also present perspectives on the use of these reactions in the syntheses of natural products or bioactive molecules. The discussion is divided in two based on the type of catalyst: metal/chiral ligand complex-based reactions and organocatalytic reactions.

## 2. Rare earth-catalyzed asymmetric diastereoselective Henry reaction

Lanthanides seldom show simple, predictable coordination chemistry because of their variable coordination number and geometry.^7^ Their coordination modes depend largely on ligand structure. Only lanthanum and neodymium have been applied so far in asymmetric diastereoselective Henry reactions.

### 2.1. Lanthanum-based catalysis

**Scheme 1:**
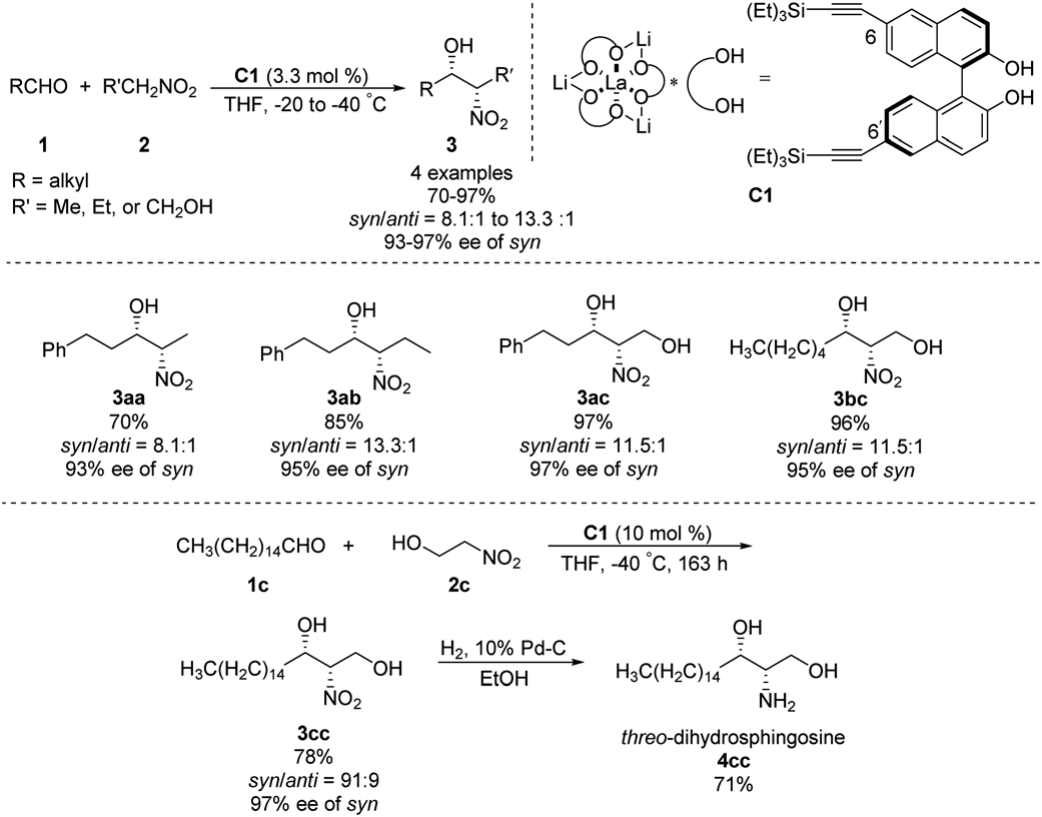
La–alkoxide complex C1 catalyzed asymmetric Henry reaction.

**Scheme 2:**
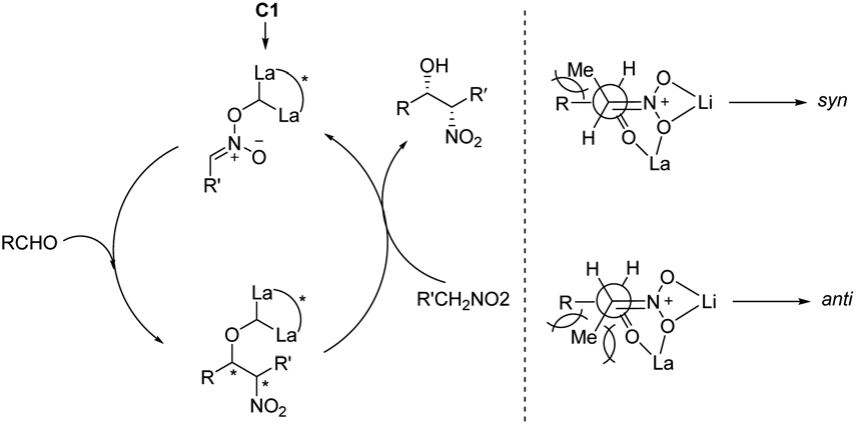
The mechanism of C1 catalyzed asymmetric Henry reaction.

In 1992, Shibasaki reported the first transition metal-catalyzed asymmetric Henry reaction.^4^ The optimal catalyst was a lanthanum–alkoxide complex C1, in which bulky TES groups at the 6,6′-positions of BINOL led to β-nitroalcohols in yields of 70–96%, a *syn*/*anti* ratio up to 92 : 8, and enantioselectivity of 93–97% ee (Scheme 1).^8^ Catalyst C1 also worked efficiently with the nitroethanol 2c bearing an alkyl aldehyde to afford the corresponding propylene glycol 3cc in good yields and a *syn*/*anti* ratio of 91 : 9. Subsequent hydrogenation reduced the nitro group to give *threo*-dihydrosphingosine 4cc in 71% yield. Mechanistic studies on the catalyst system indicated that the first step of the reaction might undergo the ligand exchange between the binaphthol and nitromethane (Scheme 2). The model proposed to explain that the *syn*-selectivity is most favorable due to steric hindrance in the bicyclic transition state *via* chelate formation which can be seen in Newman projections.

**Scheme 3:**
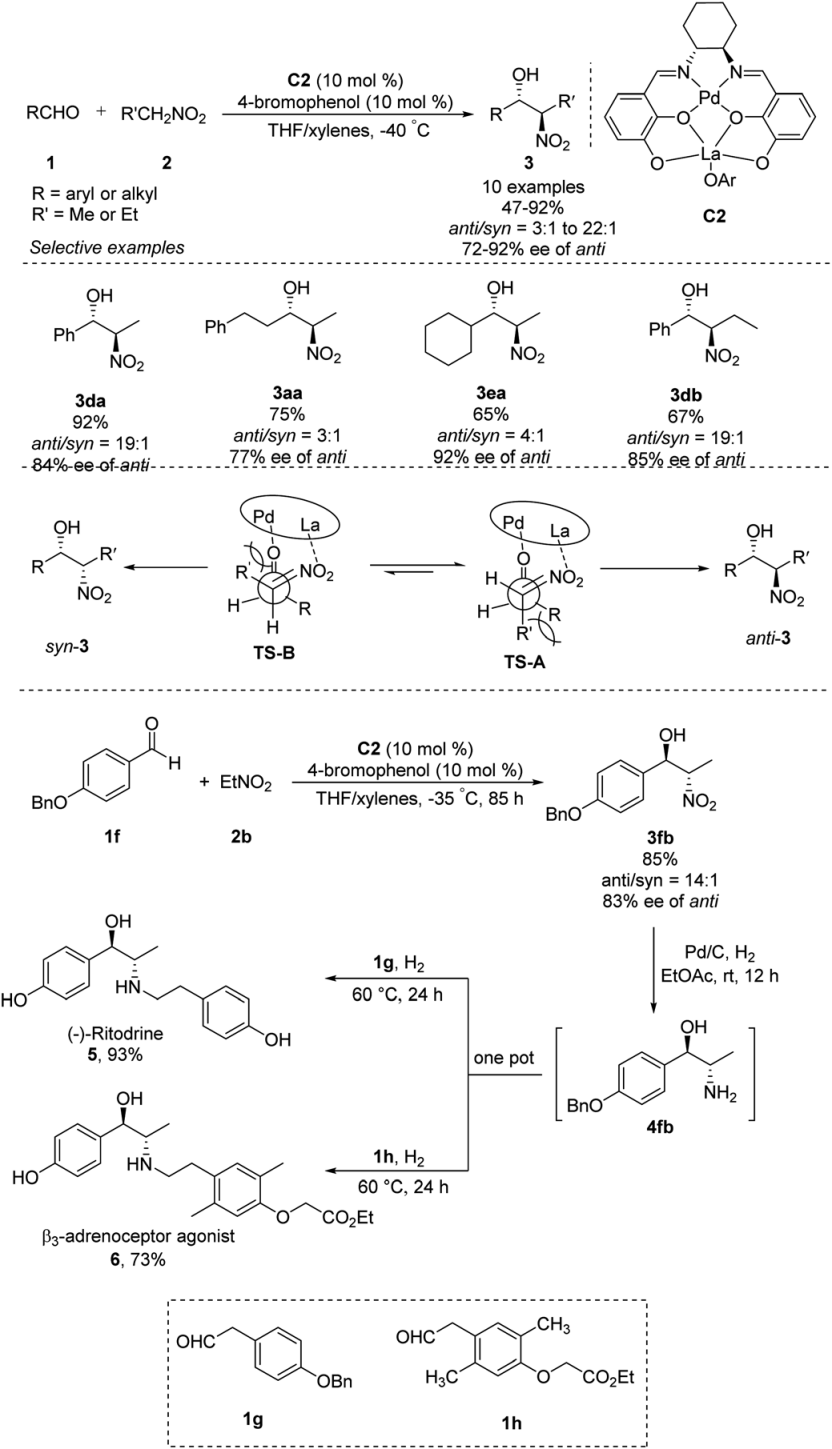
Asymmetric *anti*-selective Henry reactions catalyzed by heterobimetallic catalyst C2.

Shibasaki's group expanded the usefulness of this reaction for pharmaceutical syntheses by developing heterobimetallic Pd/La/C2 complexes that can catalyze *anti*-selective asymmetric Henry reactions of various aldehydes with nitroethane or nitropropane. In the presence of catalytic amounts of 4-bromophenol additive, products were generated in yields of 65–92% with high *anti*/*syn* ratios of 22 : 1–3 : 1 and excellent enantioselectivities of 72–92% ee (Scheme 3).^9^ The La–OAr moiety in the catalyst acts as a Brønsted base to generate a La–nitronate. Then La–nitronate reacts with the aldehyde, which is coordinated to the Pd metal center to favorably form TS-A than TS-B to avoid steric repulsion between the R′ group and the Pd/La catalyst, preferentially giving *anti*-adducts (Scheme 3).^10^ This approach generated *anti*-nitroaldol adduct 3fb, which was converted in a one-pot reaction into β-adrenoceptor agonists 5 and 6.

### 2.2. Catalysis by a neodymium–sodium heterobimetallic complex

**Scheme 4:**
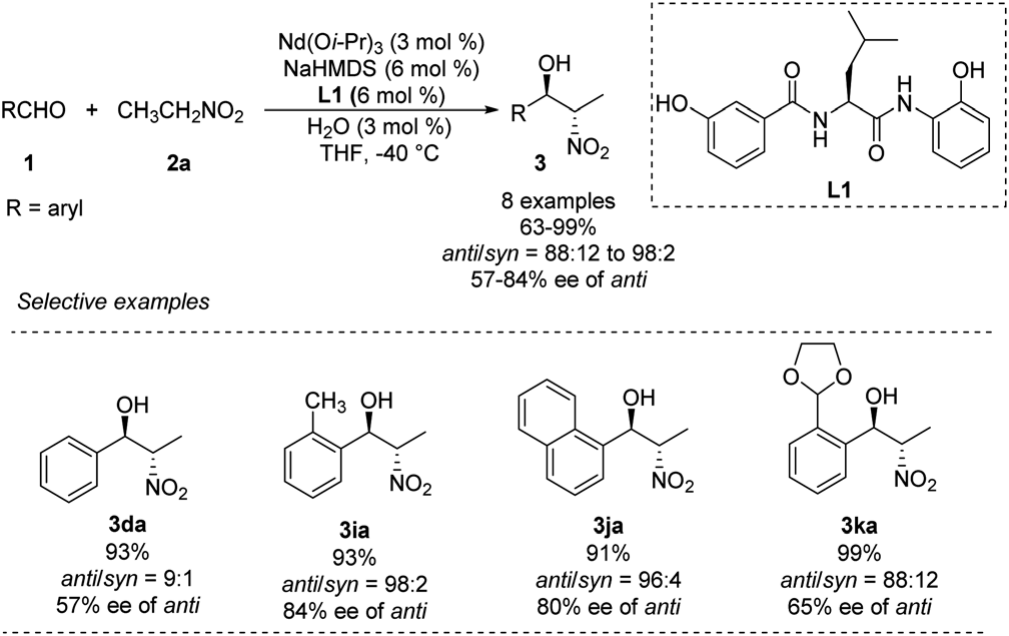
Catalytic asymmetric *anti*-selective Henry reactions with Nd/Na/L1 heterobimetallic complex.

In 2007, Shibasaki and Kumagai designed a novel lanthanum/amide complex that mimics an enzyme's structure to support asymmetric amination.^11^ Their Nd/Na/amide heterobimetallic catalyst smoothly generated *anti*-1,2-nitro alkanols with good enantioselectivities and excellent diastereoselectivities (Scheme 4).^12^ Benzaldehydes afforded the corresponding products in high yields with *anti*-selectivity, albeit only moderate enantioselectivity. Reactions of aromatic aldehydes bearing *o*-alkyl substituents proceeded smoothly with 3 mol% catalyst loading, yielding products with good diastereo- and enantioselectivities. No aliphatic aldehydes have been studied.

**Scheme 5:**
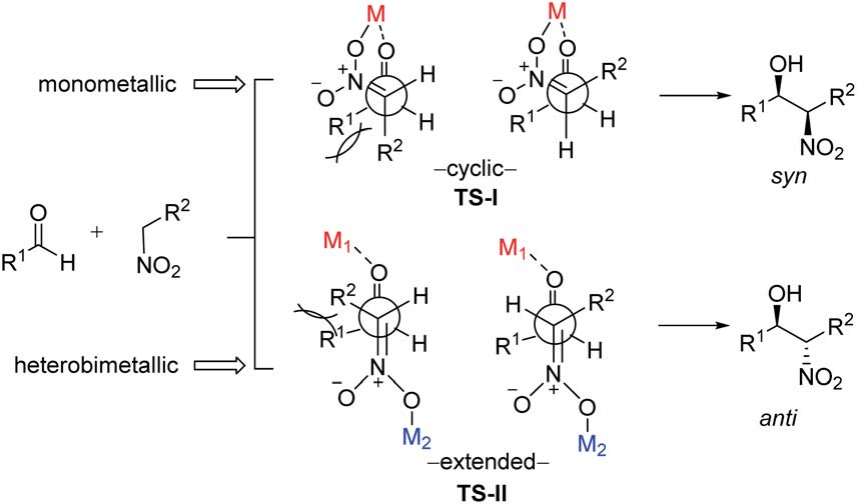
Transition state models of metal-catalyzed Henry reaction.

To account for the extremely high catalytic diastereoselectivities in the presence of heterobimetallic catalyst, Shibasaki and Kumagai proposed a transition state model for metal-catalyzed nitroaldol reactions (Scheme 5).^13^ In this mechanism, the monometallic catalyst forms the cyclic transition state TS-I when metal and oxidant chelate each other, and this transition state affords *syn* diastereomers. In contrast, chelation between a heterobimetallic catalyst and chiral amide ligand generates transition state TS-II, which prefers an extended conformation in which each metal cation works independently as a Lewis acid to activate the aldehyde and as a Brønsted base to form metal nitronate (Scheme 5). This transition state affords predominantly *anti* diastereomers, overriding the undesirable chelate formation.

**Scheme 6:**
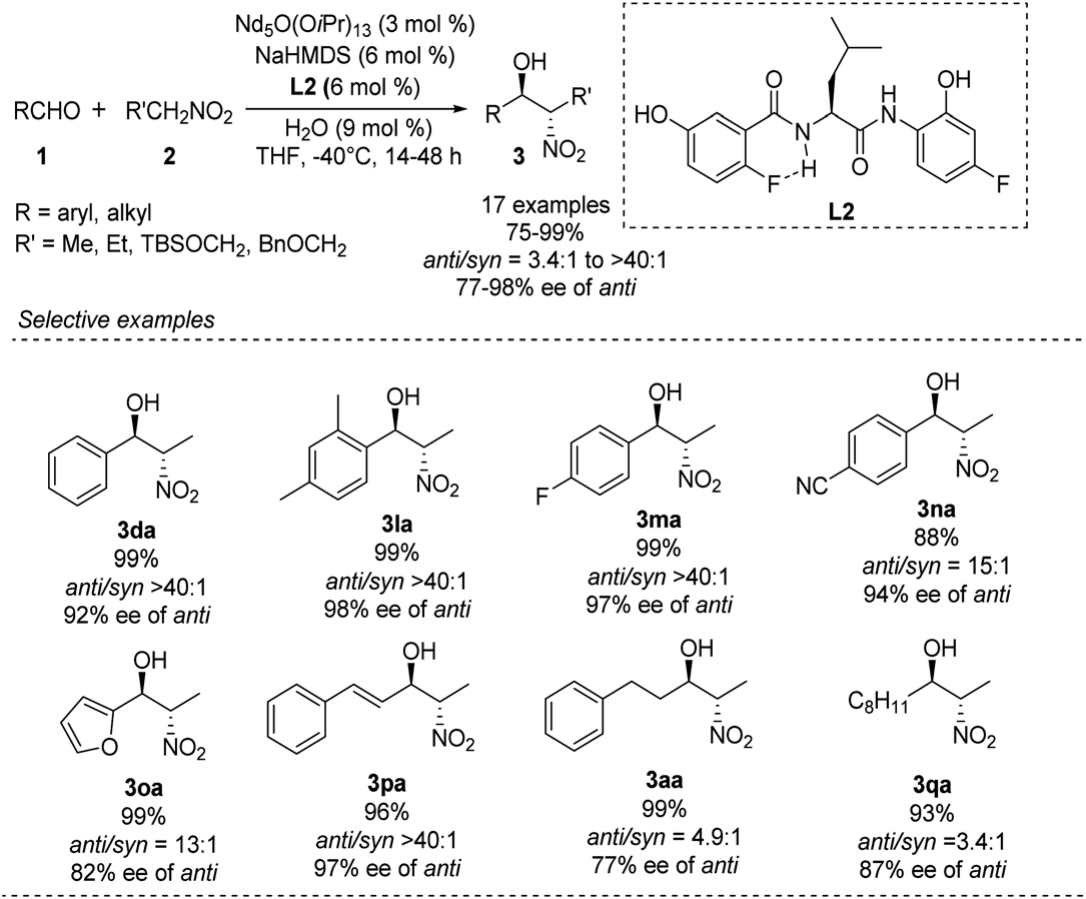
*o*-Fluorobenzamide L2 as chiral ligand of the asymmetric *anti*-selective Henry reaction.

**Scheme 7:**
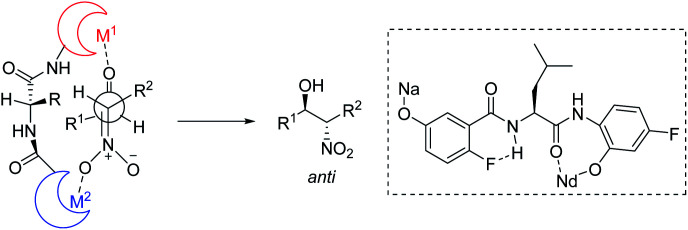
Amide backbone as a platform for bimetallic complex.

Kumagai and Shibasaki improved the *anti*-selectivity and enantioselectivity of catalytic asymmetric Henry reactions by using a fluorine-substituted chiral amide ligand L2 with their Nd/Na heterobimetallic complex (Scheme 6).^14^ The corresponding products formed with nearly perfect *anti*-selectivity, which the researchers attributed to (1) a C–F⋯H–N intramolecular hydrogen bond in the *o*-fluorobenzamide, which may restrict rotation of the C–C bond; (2) the influence of the fluorine substituent on the electronic properties of the aminophenol moiety (Scheme 7). Regardless of the reasons, the researchers found that aliphatic aldehydes led to much lower *anti*-selectivity than aromatic aldehydes.

**Scheme 8:**
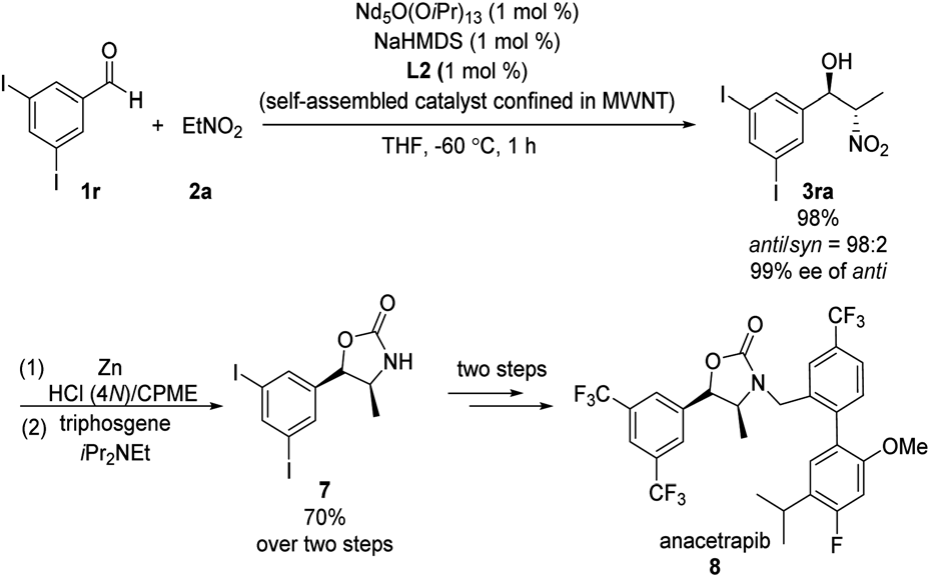
Enantioselective synthesis of anacetrapib.

Kumagai and Shibasaki found that immobilizing their Nd/Na heterobimetallic catalyst on an entangled multiwalled carbon nanotube substantially increased its efficiency and facilitated its reuse.^15^ Using this self-assembling catalyst system, they concisely prepared anacetrapib (8) enantioselectively (Scheme 8). The catalyst promoted the reaction of diiodobenzaldehyde 1r with nitroethane 2a, providing *anti*-β-nitroethanol 3ra in excellent yield as well as excellent diastero- and enantioselectivity. A further four steps completed the synthesis of 8. In later work, Shibasaki replaced NdO_1/5_(O^i^Pr)_13/5_/NaHMDS with bench-stable, inexpensive NdCl_3_·6H_2_O/NaO^*t*^Bu.^16^

**Scheme 9:**
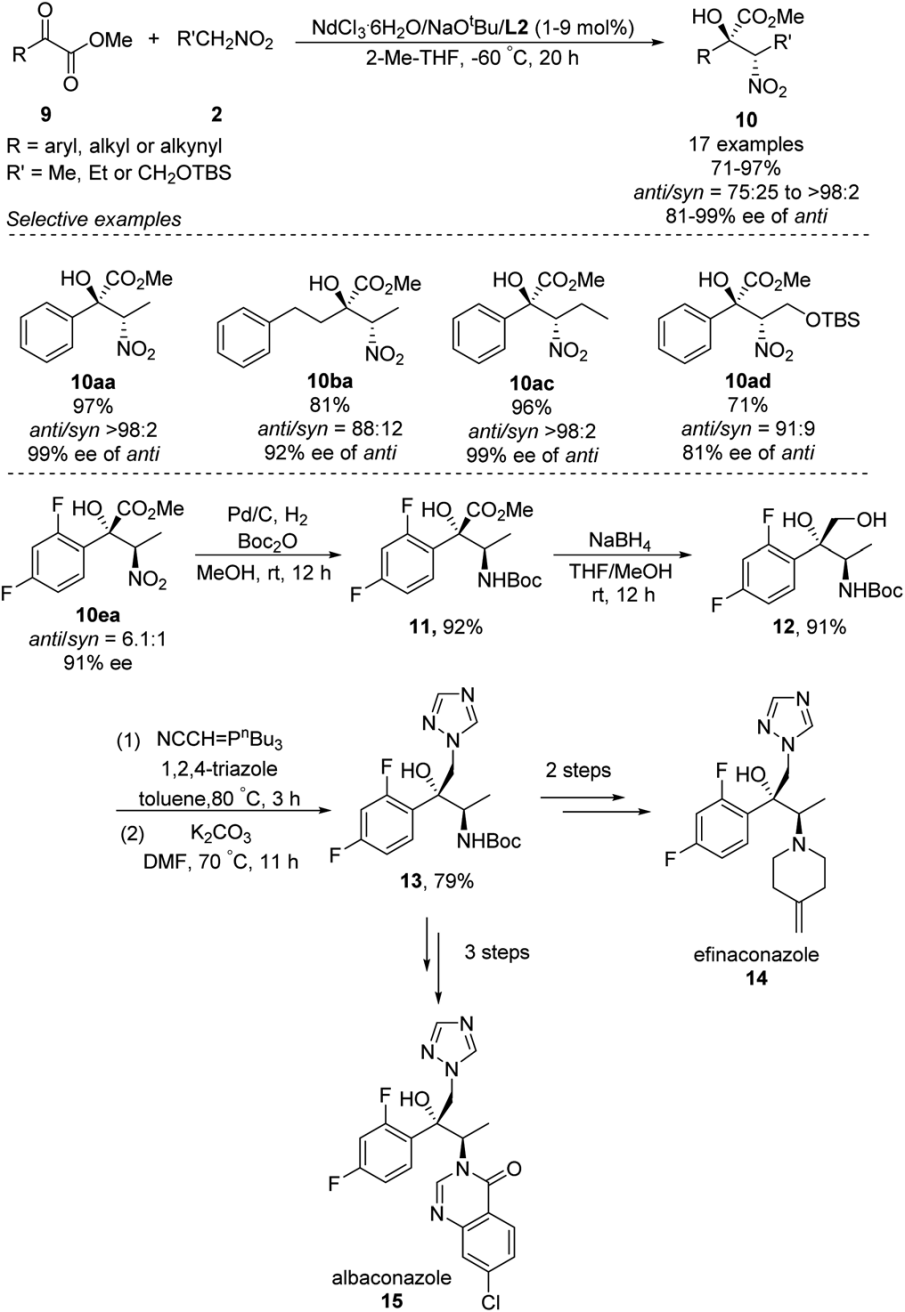
*Anti*-selective asymmetric Henry reaction of α-keto esters.

In 2018, Kumagai and Shibasaki extended Nd/Na heterobimetallic catalysts to reactions between α-keto esters and nitroalkanes (Scheme 9).^17^ In this approach, a range of aryl α-keto esters afforded α-nitro tertiary alcohols in good to excellent yields as well as diastero- and enantioselectivities. The solvent 2-Me-THF gave better stereoselectivity than THF. The reaction also tolerated alkyl and alkynyl α-keto esters, albeit with modest diastereo- and enantioselectivities. The researchers exploited the *anti*-selectivity of their asymmetric Henry reaction to streamline the stereoselective synthesis of the commercial antifungal agents efinaconazole (14) and albaconazole (15). In the shared starting pathway, the nitro group in α-keto esters 10ea was reduced and the compound protected with a Boc group, giving methyl ester 11 in 92% yield. Reduction with NaBH_4_ gave diol 12, and introduction of 1,2,4-triazole afforded the key intermediate 13, from which two further steps generated efinaconazole (14) or three further steps generated albaconazole (15).

**Scheme 10:**
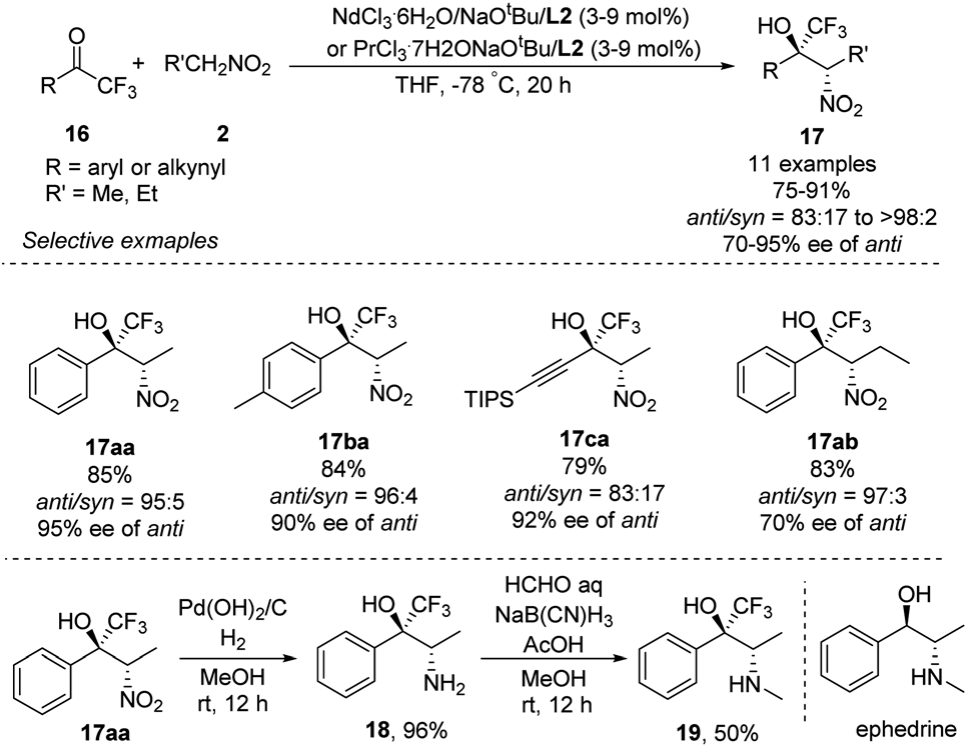
Asymmetric Henry reactions of trifluoromethyl ketones.

Highly *anti*-selective Henry reactions of various trifluoromethyl ketones with nitroethane and 1-nitropropane were also achieved using heterobimetallic Nd–Na–L2 or Pr–Na–L2 complex (Scheme 10).^18^ Nitroethane led to CF_3_-appended *vic*-nitroalkanols in *anti*/*syn* ratios up to 98 : 2 and 95% ee. Nitropropane, in contrast, led to much lower enantioselectivity of 70% ee. This reaction was also able to generate CF_3_-appended ephedrine 19.

While these impressive results highlight the ability of rare earth metals to support efficient enantio- and diastereoselective Henry reactions, such metals are scarce and expensive. This has led researchers to search for more accessible and inexpensive metal catalysts.

## 3. Copper-catalyzed asymmetric diastereoselective Henry reaction

Copper is abundant, shows low toxicity and can form stable chiral metal complexes with ligands containing nitrogen- and oxygen.^19^ It is no surprise, then, that since Jørgensen's groundbreaking work in 2001,^20^ numerous asymmetric diastereoselective Henry reactions have been developed using chiral copper-based catalysts and various chiral ligands such as imidazolines, Schiff bases, tetrahydrosalens, amino alcohols and diamines.

### 3.1. Chiral imidazoline ligands

**Scheme 11:**
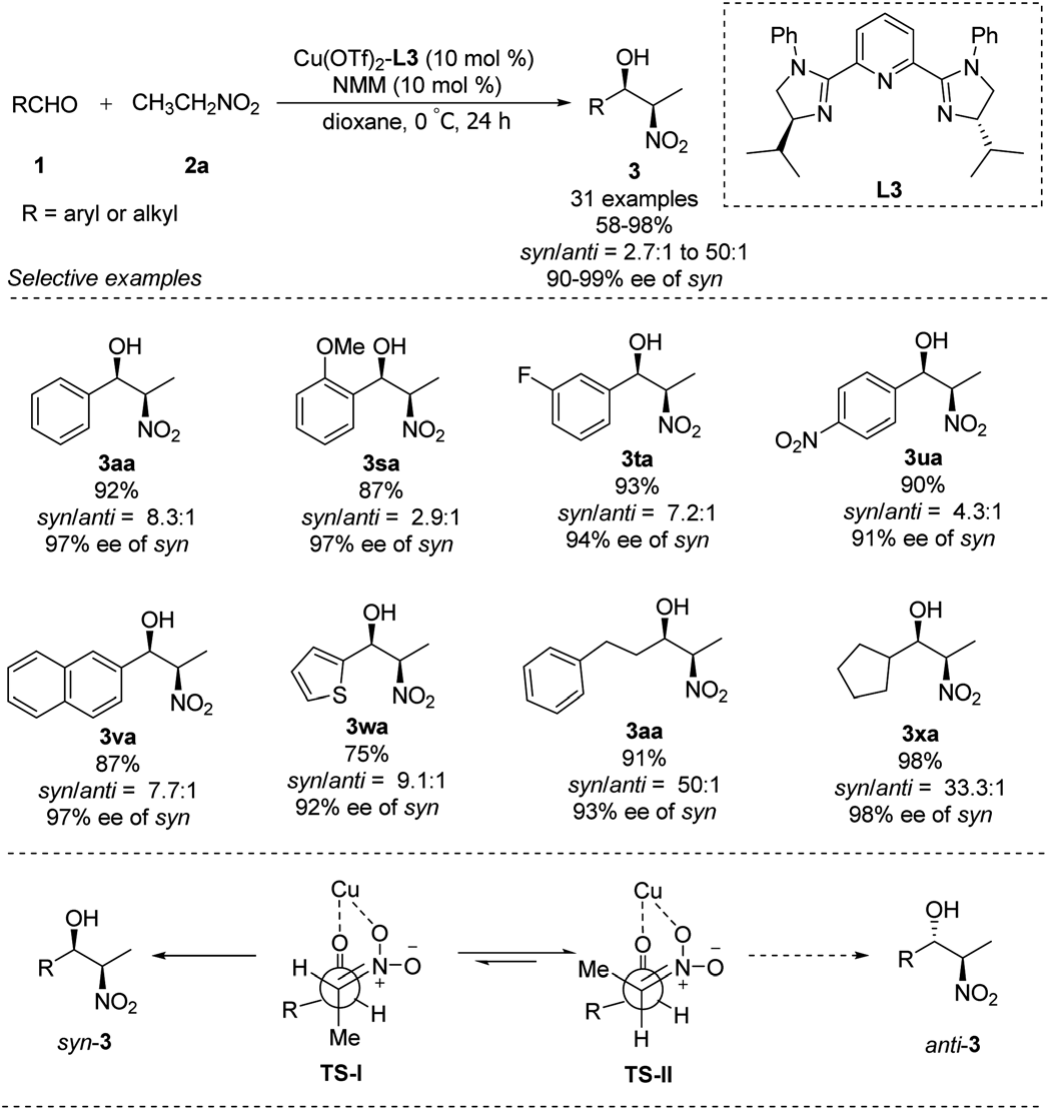
You's asymmetric diastereoselective Henry reaction.

In 2007, You replaced the oxygen atom of oxazoline with nitrogen to generate tridentate imidazoline ligand L3.^21^ The complex of Cu(OTf)_2_–L3 supported enantioselective Henry reactions in the presence of catalytic amounts of Et_3_N. In reactions using nitroethane as nucleophile, this catalyst demonstrated good synthetic potential, that aromatic, aliphatic and even heterocyclic aldehydes are well tolerated (Scheme 11).^22^ Surprisingly, using *N*-methylmorpholine as base generated the desired adducts with a *syn*/*anti* ratio up to 50 : 1 and enantioselectivity up to 99% ee. In this process, TS-I is most favoured and results in *syn* product, because of the repulsion between the methyl group of nitroethane and the isopropyl group of the catalyst in Ts-II, which will lead to the *anti* product (Scheme 11).

### 3.2. Chiral Schiff-base ligands

**Scheme 12:**
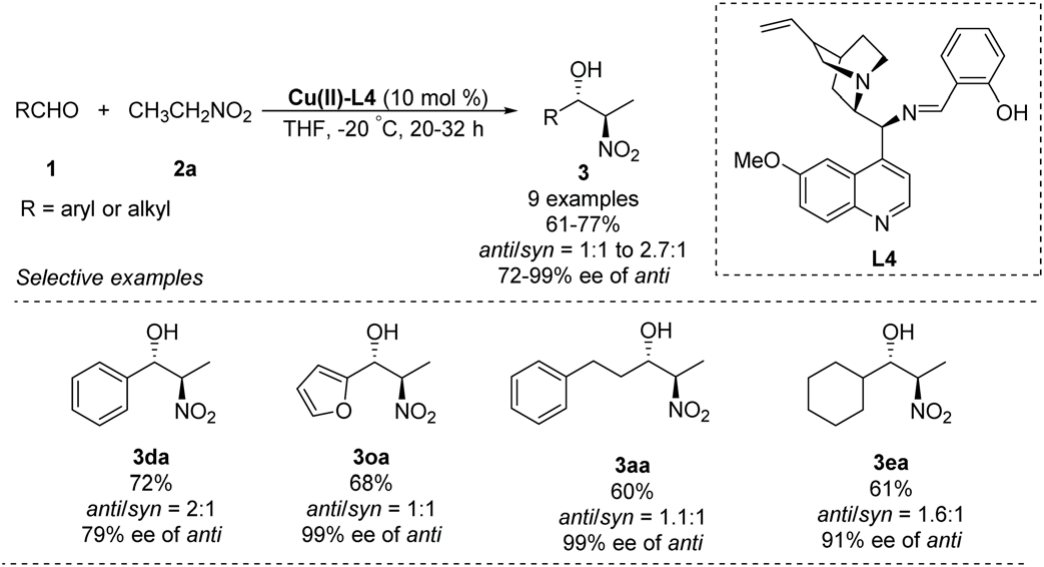
Cu–cinchona alkaloid complex catalyzed asymmetric Henry reaction.

A chiral Schiff-base ligand L4, derived from cinchona alkaloid, supported smooth asymmetric Henry reactions between various aldehydes and nitroethane.^23^ The corresponding products were obtained in yields around 70% with enantioselectivities up to 99% ee, but *anti*/*syn* ratios up to only 2.7 : 1 (Scheme 12).

### 3.3. Chiral tetrahydrosalen ligands

Chiral salen-type ligands have proven useful in a variety of asymmetric metal-catalyzed reactions.^24^ In Henry reactions, chiral tetrahydrosalen ([H_4_]salen) ligands produce strong asymmetry by increasing the basicity and framework flexibility of the nitrogen atom.^25^

**Scheme 13:**
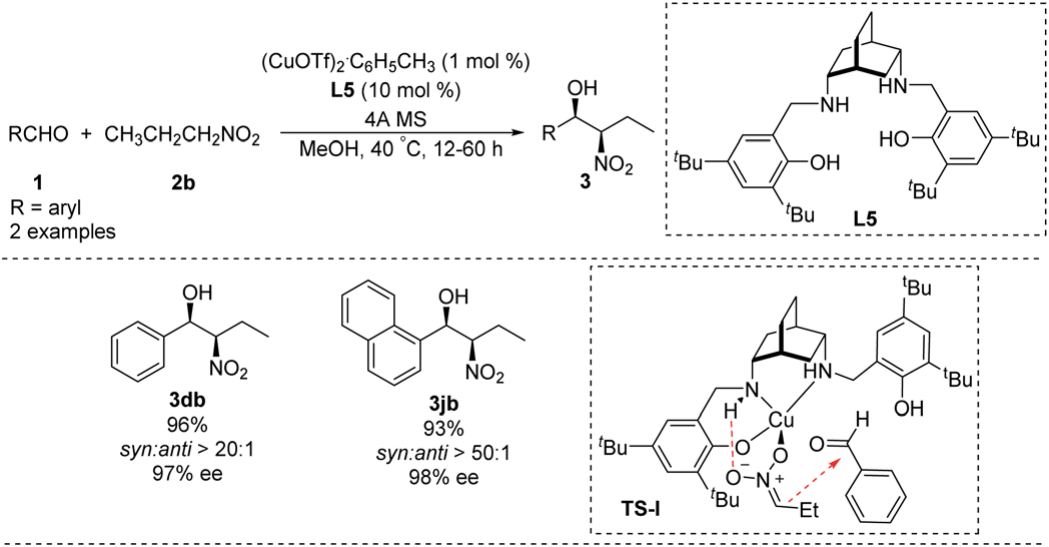
Asymmetric *syn*-selective Henry reaction catalyzed by copper(i)–[H_4_] salen complex.

In 2012, White synthesized chiral [H_4_]salen ligand L5 from *cis*-2,5-diaminobicyclo[2.2.2]octane (Scheme 13)^26^ and used it to conduct a highly enantio- and diastereoselective copper(i)-catalyzed Henry reaction. Reaction of benzaldehyde and 1-naphthaldehyde with nitropropane in the presence of Cu(i) and L5 strongly favoring the *syn* product 3 which was formed in high enantiomeric excess. A transition state TS-I rationalizing this outcome is proposed. In this model, N–H hydrogen bond with the nitronate leads to high enantioselectivity. In addition, the copper complexed nitronate of nitropropane in TS-I has a (*Z*) configuration with attack occurring at the *si* face of the aldehyde carbonyl (Scheme 13). While the applicability of this approach is limited by the expense of the metal and complicated preparation of ligand L5.

**Scheme 14:**
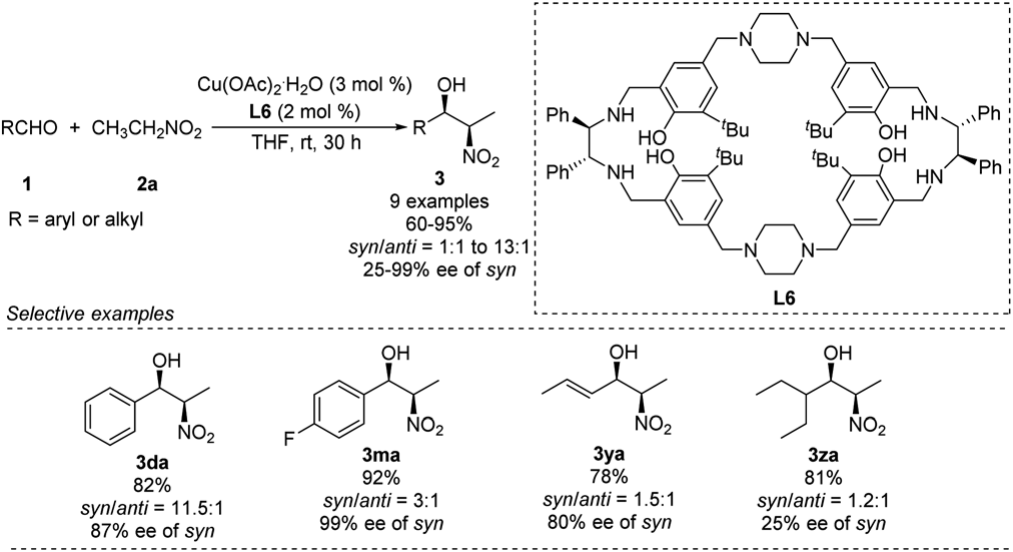
Asymmetric *syn*-selective Henry reaction catalyzed by Cu(ii)–L6 complex.

Kureshy developed Cu–L6 complexes to catalyze diastereoselective Henry reactions (Scheme 14).^27^ Benzaldehyde reacted with nitroethanol to give the desired product in 82% yield with a *syn*/*anti* ratio of 92 : 8. However, the reaction did not work well with aliphatic and aromatic aldehydes bearing electron-withdrawing substitutions. The researchers were able to use the catalyst more than 8 times without significant loss of performance.

### 3.4. Chiral amino-alcohol ligands

**Scheme 15:**
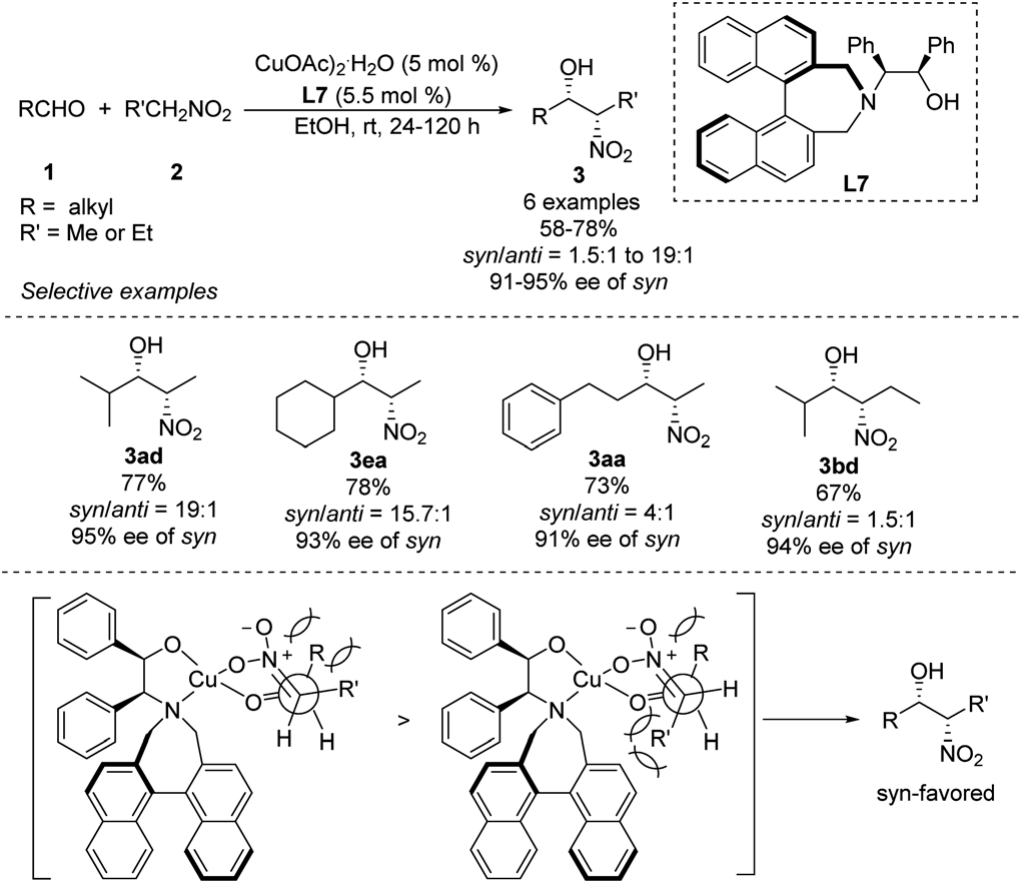
Asymmetric *syn*-selective Henry reaction catalyzed by amino alcohol ligand L7.

Early in 2011, Lu derived amino alcohol L7 from 1,1-binaphthylazepine and demonstrated its efficiency as a chiral ligand in Cu-catalyzed asymmetric Henry reactions (Scheme 15).^28^ In this catalytic system, aliphatic aldehydes showed better enantio- and diastereoselectivities than aromatic aldehydes. For example, isobutyraldehyde reacted with nitroethane to give the corresponding product in 77% yield with a *syn*/*anti* ratio of 95 : 5, and the *syn*-adduct showed enantioselectivity up to 95%. Under the same conditions, aldehydes reacted with nitropropanes to give products in *syn*/*anti* ratios of only 61 : 39. The reaction model proposed to explain the steric hindrance of binaphthylazepine in the L7 could result in higher stereocontrol.

**Scheme 16:**
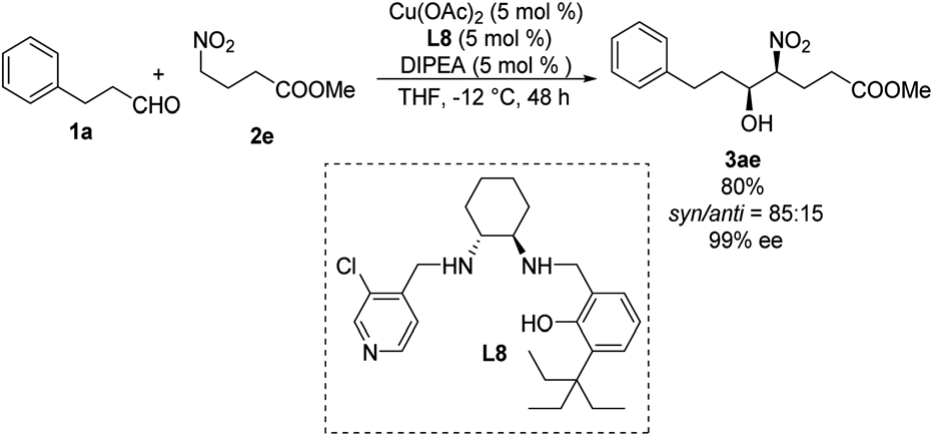
Asymmetric *syn*-selective Henry reaction catalyzed by *C*_1_-symmetrical amino-alcohol ligand L8.

Systematic screening of a library of *C*_1_-symmetrical amino-alcohol compounds showed that those bearing a pyridine group and a phenol substituted with a bulky alkyl group were the best ligands in diastereoselective Henry reactions.^29^ In the presence of 5 mol% of Cu(OAc)_2_–L8 as catalyst and 5 mol% diisopropylethylamine as base, the Henry reaction of 3-phenylpropionaldehyde 1a and methyl 4-nitrobutyrate 2e furnished the desired products with a *syn*/*anti* ratio of 85 : 15 (Scheme 16). Using aromatic aldehydes substantially reduced the *syn*/*anti* ratio to 1.7 : 1.

**Scheme 17:**
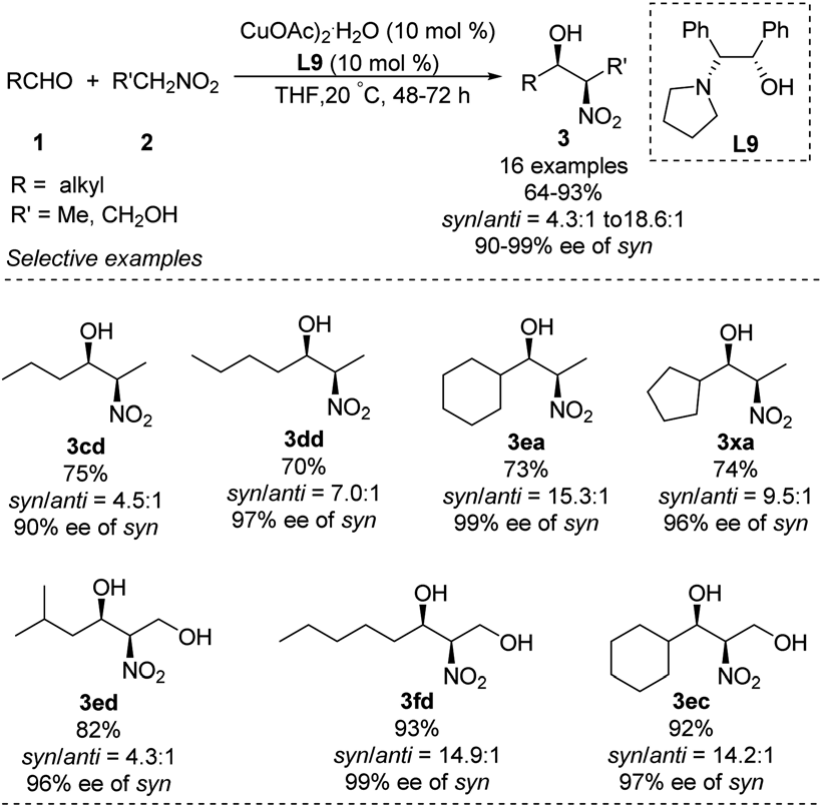
*Syn*- and enantioselective Henry reactions of aliphatic aldehydes.

**Scheme 18:**
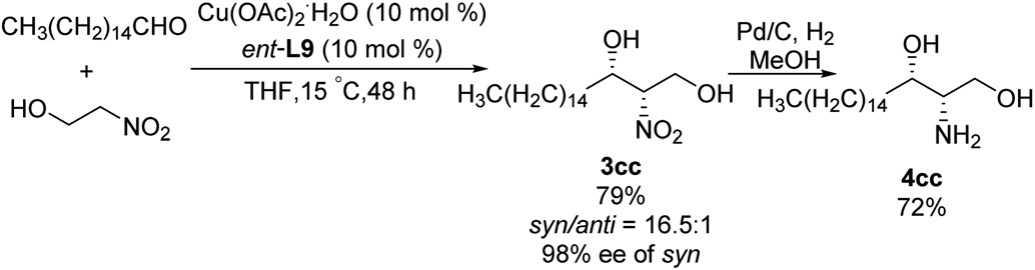
Synthesis of safingol.

Chen developed an amino alcohol copper(ii) catalyst (Cu–L9) for *syn*- and enantioselective Henry reactions of aliphatic aldehydes with nitroethane (Scheme 17).^30^ The desired products were obtained with very good enantioselectivity and *syn*/*anti* ratios up to 18.6 : 1. In contrast, 4-chlorobenzaldehyde generated product in a *syn*/*anti* ratio of only 2.9 : 1 under optimal conditions. Using 2-nitroethanol as nucleophile increased the *syn*/*anti* ratio, presumably because the hydroxyl group formed additional intermolecular hydrogen bonds and thereby stabilized transition states. This approach allowed the preparation of safingol (4cc) in only two steps with 57% overall yield (Scheme 18).^31^

**Scheme 19:**
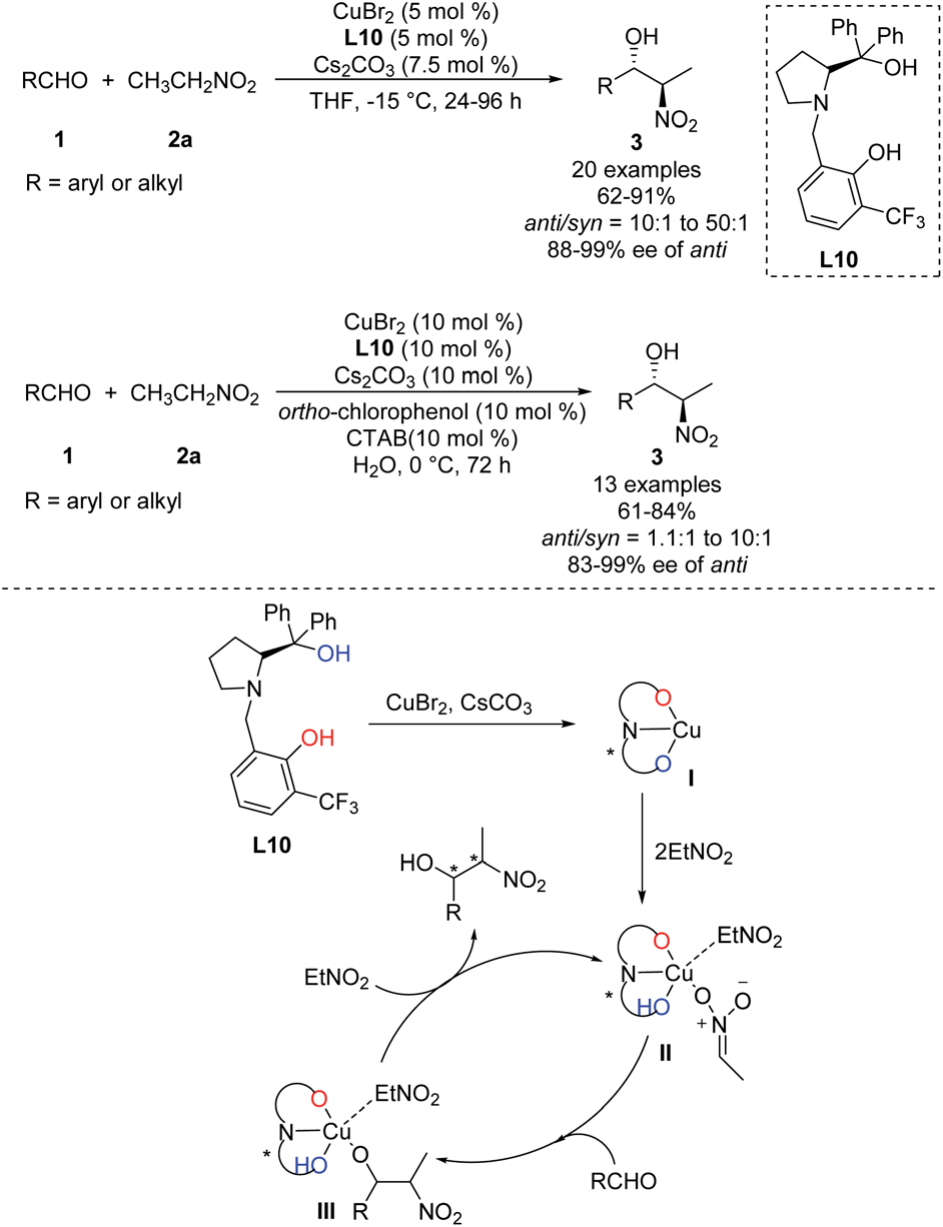
*Anti*-selective asymmetric Henry reactions in the presence of water.

In 2011, Wang achieved one of the few monometal-catalyzed *anti*-selective asymmetric Henry reactions ever reported. They conducted the reaction in organic solvents and water using the catalyst Cu–L10 and phase-transfer catalyst Bu_4_NBr.^32^ They achieved good *anti*-selectivity and excellent enantioselectivities, even 99% ee in water (Scheme 19).^33^ The transition metal model exhibited that copper complex was bonded to one molecule of nitronate and coordinated with one molecule of EtNO_2_ in the intermediate II, then only metal–nitronate worked with aldehyde to give *anti*-selectivity Henry reactions. Therefore, this reaction requires excess EtNO_2_(Scheme 19). Further analysis showed that diastereoselectivity did not depend on 4-*tert*-butylphenol or Bu_4_NBr, and that phenol may facilitate proton transfer by functioning as a weak acid.^34^

**Scheme 20:**
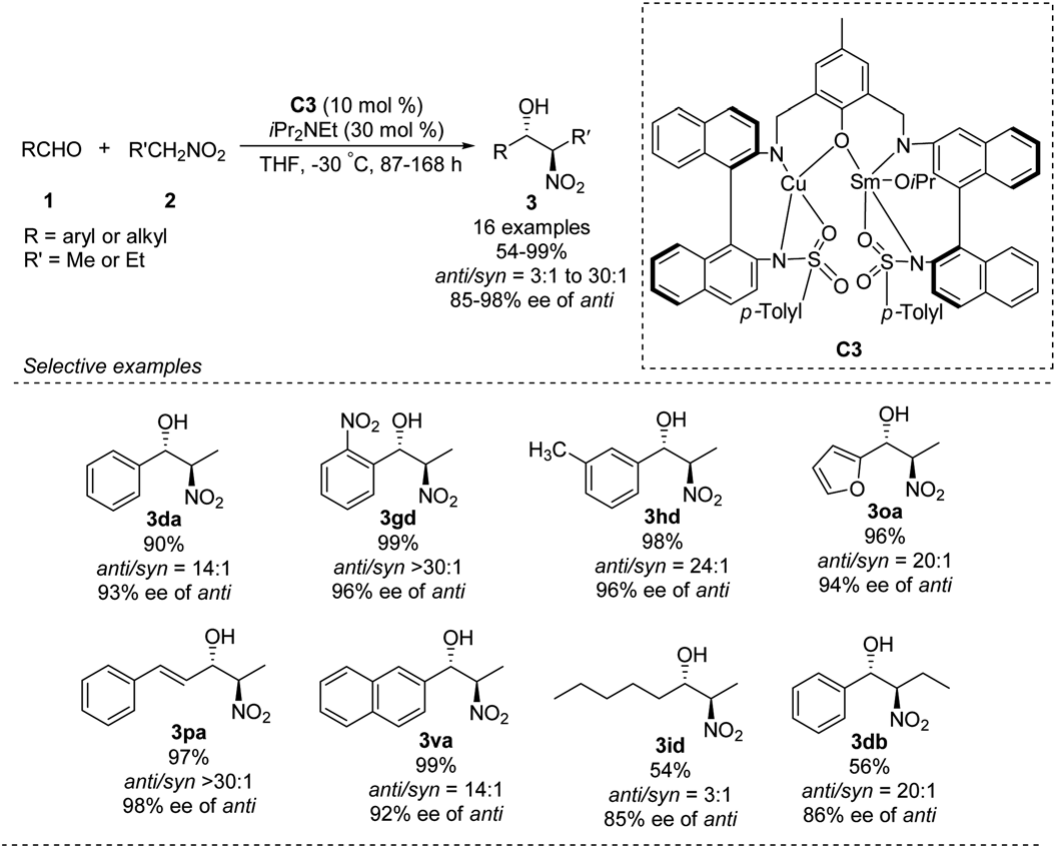
*Anti*-selective asymmetric Henry reaction catalyzed by heterobimetallic Cu/Sm/aminophenol sulfonamide complex.

In 2016, Zhou prepared the novel heterobimetallic Cu/Sm/aminophenol sulfonamide complex C3 in one pot and used it to achieve an *anti*-selective asymmetric Henry reaction (Scheme 20).^35^ Aryl aldehydes substituted with electron-donating or -withdrawing substituents afforded the products in up to 99% yield with *anti*/*syn* ratios > 30 : 1 and enantioselectivity of 98% ee. The reaction with benzaldehyde also proceeded with 1-nitropropane as nucleophile. As showed in the report, aromatic aldehydes led to higher diastereoselectivity.

### 3.5. Chiral diamines ligands

**Scheme 21:**
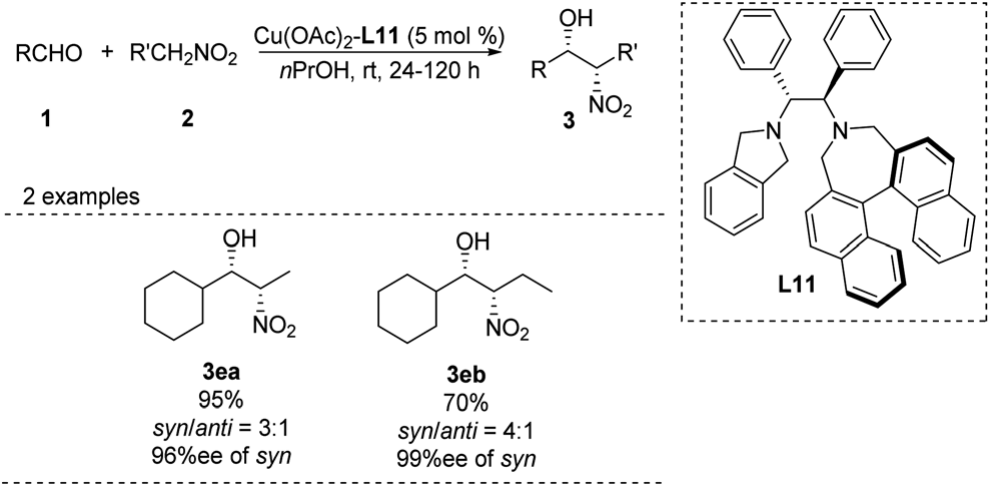
Arai's asymmetric Henry reaction.

In 2006, Arai catalyzed the Henry reaction using a *C*_2_-symmetric diamine catalyst,^36^ whose usefulness is limited by the air sensitivity of Cu(i) and the hygroscopic cyclohexyl-1,2-diamine ligand. To overcome these drawbacks, the researchers replaced the binaphthyl azepine ring in the ligand with a simple isoindoline to generate ligand L11.^37^ In the presence of 5 mol% of Cu(OAc)_2_–L11 complex, the adduct was obtained in >99% yield with 98% ee at room temperature (Scheme 21). The same catalyst supported other *syn*-selective Henry reactions, which afforded both diastereomers with excellent enantiomeric excess.

**Scheme 22:**
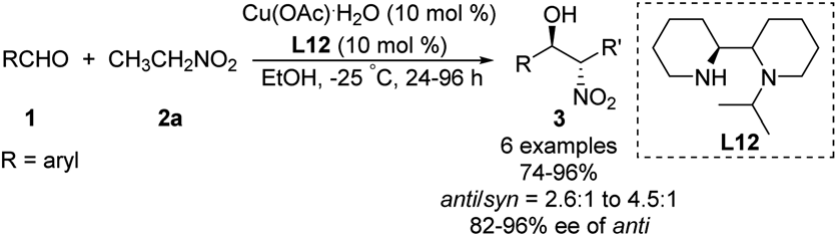
Cu(ii)–L12 catalyzed Henry reactions.

Kanger reported an asymmetric Henry reaction using L12 as the chiral ligand (Scheme 22).^38^ The desired Henry adducts were efficiently obtained at low temperature after reasonable reaction times with enantioselectivities up to 96%. Using other nitroalkyl compounds as ligands gave lower *anti*/*syn* ratios from 2.6 : 1 to 4.5 : 1.

**Scheme 23:**
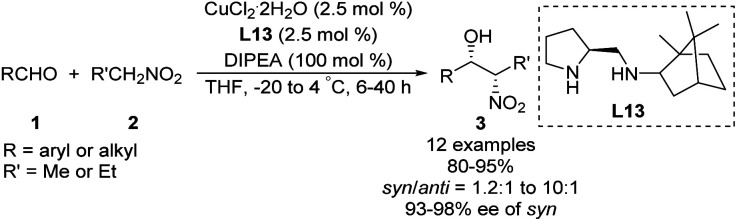
*Syn*-selective asymmetric Henry reaction catalyzed by L13.

Zhang and Guo developed the *C*_1_-symmetric chiral diamine L13 as an efficient ligand for the copper-catalyzed asymmetric nitroaldol reaction.^39^ Their catalyst system supported the reaction of nitroethane or 1-nitropropane with 2-nitroethylbenzene to afford products with excellent enantioselectivities and moderate to good diastereoselectivities (Scheme 23). Due to its stability, ligand L13 was recovered in good yield without loss of catalytic performance *via* simple aqueous acid/base workup.

**Scheme 24:**
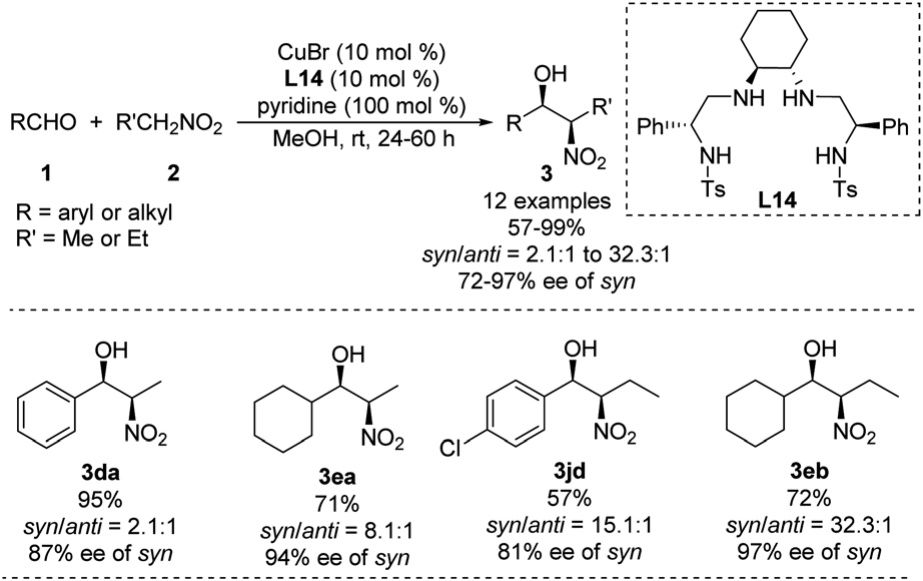
*Syn*-selective asymmetric Henry reaction catalyzed by Cu–L14.

Using a chiral bis(sulfonamide)-diamine skeleton and nitromethane as nucleophile, Wan achieved copper-catalyzed enantioselective Henry reactions giving good yields and high enantioselectivities.^40^ A scalable version of the reaction gave adducts in up to 99% yield with a *syn*/*anti* ratio of 32.3 : 1, and 97% ee of the *syn* adduct (Scheme 24).^41^ This *syn*-selectivity appears to depend on pyridine, and base additives increase catalyst reactivity.^42^ Future work should examine whether other complex nitroalkanes can support high diastereo- and enantioselectivities in this reaction.

**Scheme 25:**
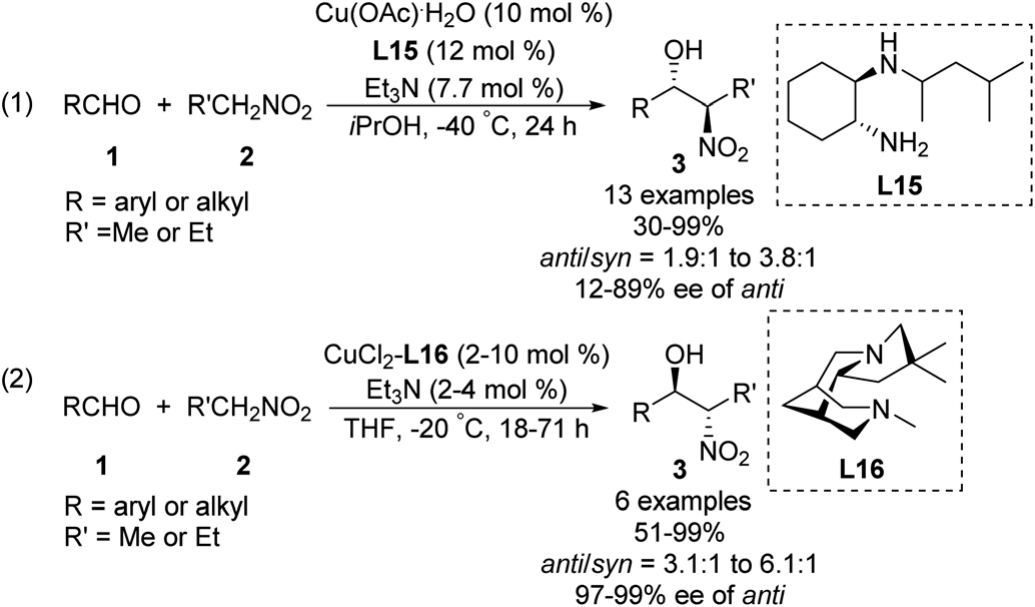
*Anti*-selective asymmetric Henry reaction catalyzed by Cu–L15 and Cu–L16.

Some of the few monometal-catalyzed *anti*-selective asymmetric Henry reactions were achieved using Gou's chiral *N*-monoalkyl cyclohexane-1,2-diamine ligand L15 ^43^ and Breuning's chiral bispidine ligand L16.^44^ These reactions gave products with *anti*/*syn* ratios of 6.1 : 1 and enantioselectivity above 90% ee (Scheme 25).

### 3.6. Other type of chiral ligands

**Scheme 26:**
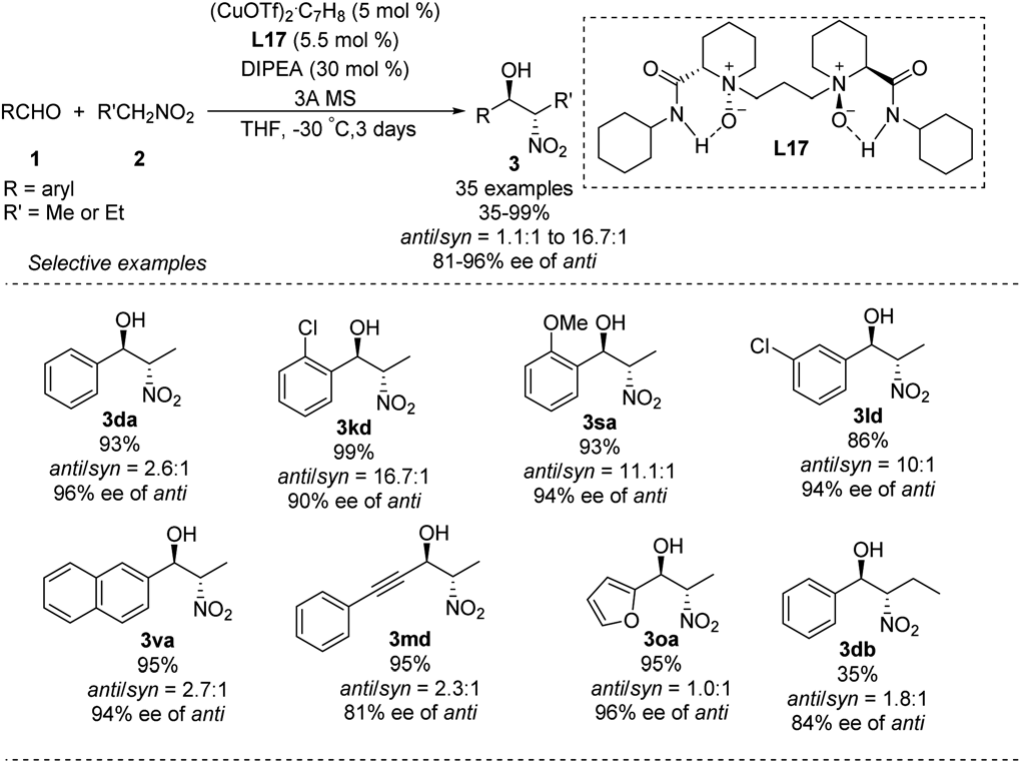
*N*,*N*-dioxide ligands applied to the Cu-catalyzed asymmetric *anti*-selective Henry reactions.

*N*,*N*-dioxide/metal complexes have been used to catalyze a number of enantioselective reactions.^45^ In 2007, Feng reported enantioselective Henry reactions catalyzed by *N*,*N*′-dioxide-Cu(i).^46^ These ligands also proved efficient for asymmetric *anti*-selective Henry reactions of nitroethane with aromatic aldehydes, which generated the corresponding products in good yields with moderate to excellent dr values (Scheme 26).^47^ However, these results required low reaction temperatures and long reaction times. Naphthaldehyde, α,β-unsaturated aldehyde and heteroaromatic aldehyde proceeded well to afford the nitroaldol products in good yields but with poor *anti*/*syn* ratio. In addition, the poor reactivity of 1-nitropropane was observed *via* its steric hindrance.

**Scheme 27:**
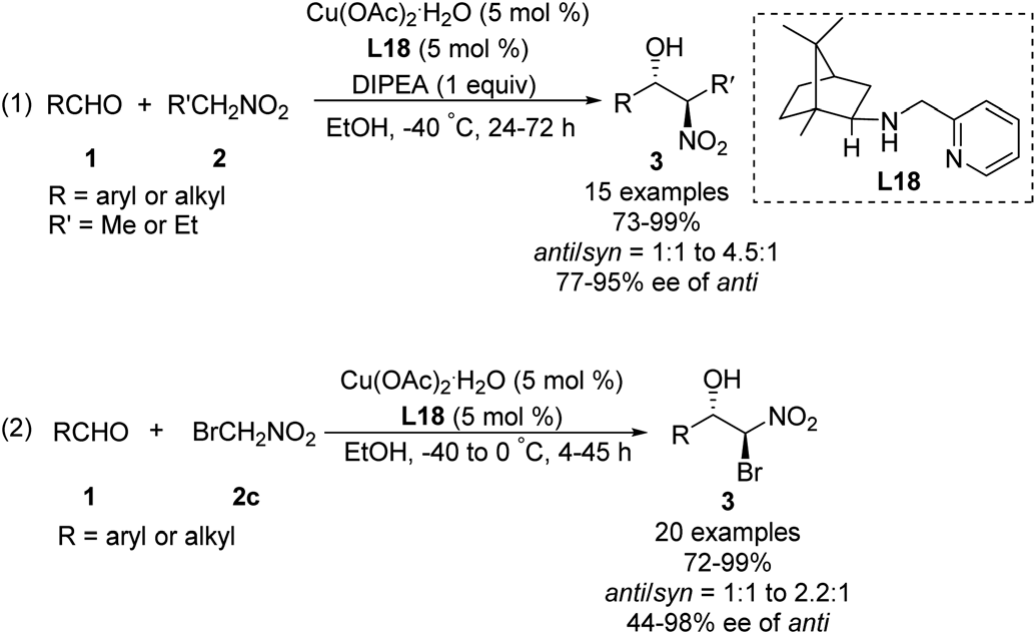
Chiral amino-pyridine ligand L18 applied to the Cu-catalyzed asymmetric Henry reactions.

In 2008, Blay and Pedro reduced the imine bond of iminopyridine to synthesize a more flexible chiral aminopyridine ligand L18.^48^ In the presence of 5 mol% of this ligand, 1.0 equiv. of DIPEA and 5 mol% of Cu(OAc)_2_·H_2_O, various aldehydes reacted smoothly with nitroethane and bromonitromethane^49^ to give the expected products in yields up to 99%, enantioselectivities up to 98% ee and diastereoselectivities up to 82 : 18 (Scheme 27).

## 4. Cobalt-catalyzed asymmetric diastereoselective Henry reaction

Optically active ketoiminatocobalt complexes were originally employed as chiral Lewis acid catalysts in enantioselective hetero Diels–Alder reactions^50^ and carbonyl-ene reactions.^51^

**Scheme 28:**
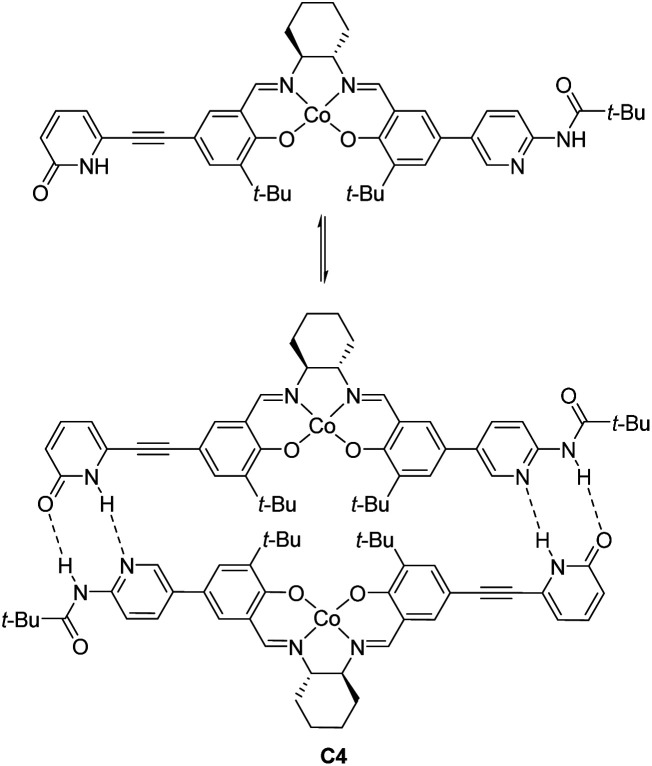
Hong's self-assembled catalyst.

In 2008, Hong revealed that self-assembled dinuclear cobalt(ii)–salen catalyst C4 promoted cobalt-catalyzed asymmetric *anti*-selective Henry reactions (Scheme 28).^52^ Dimers self-assembled from 2-pyridone and aminopyridine as the hydrogen-bonding pair.

Hong went on to develop second-generation catalysts that self-assembled through urea–urea hydrogen bonding,^53^ such as the [(bisurea–salen)Co] catalyst C5.^54^ The NH moiety of urea was critical for yield and stereoselectivity: both parameters decreased when the catalyst was replaced by unfunctionalized [(salen)Co^III^] catalyst C6 or methyl-functionalized catalyst C7 (Table 1). The author proposed that the [(bisurea–salen)Co] catalyst might enable the antiparallel transition state for the Henry reaction, either by bimetallic dual activation (TS-I) or by H-bond/metal bifunctional activation (TS-II) (Table 1).

**Table 1:** Self-assembled [(bisurea–salen)Co] catalyzed asymmetric Henry reaction

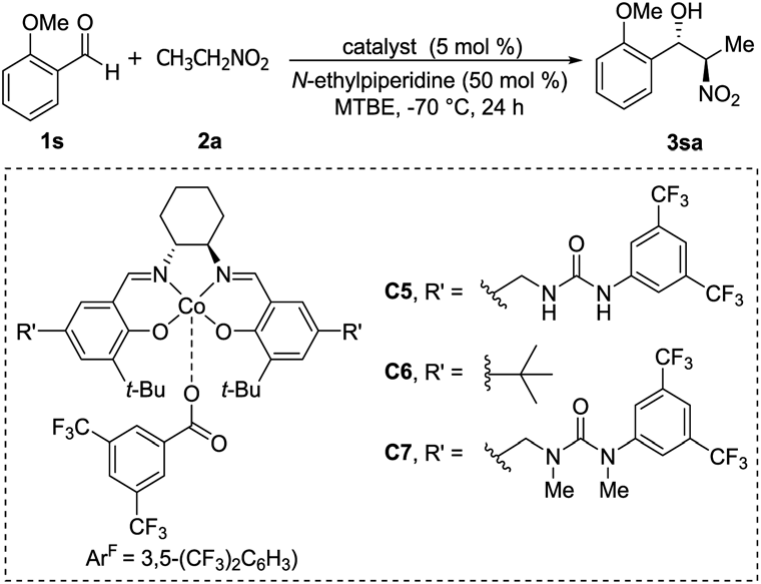			
Catalyst	Yield (%)	*Anti*/*syn*	ee of *anti* (%)
C5	84	48 : 1	96
C6	30	3 : 1	78
C7	14	4 : 1	85
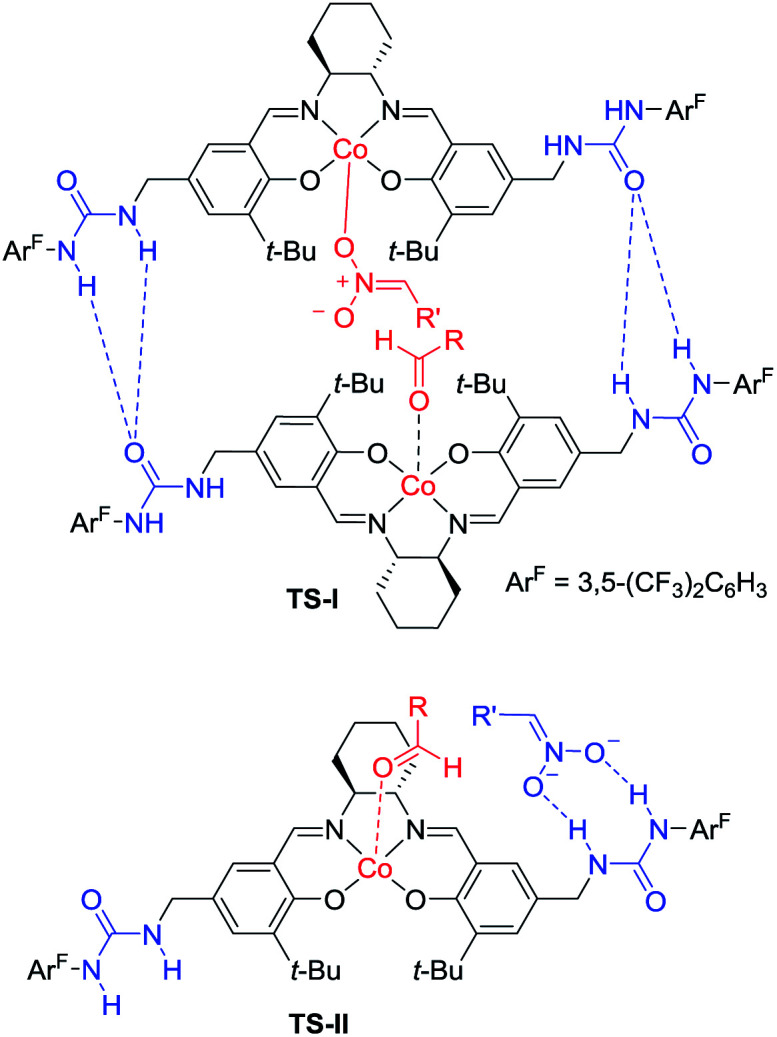			

**Scheme 29:**
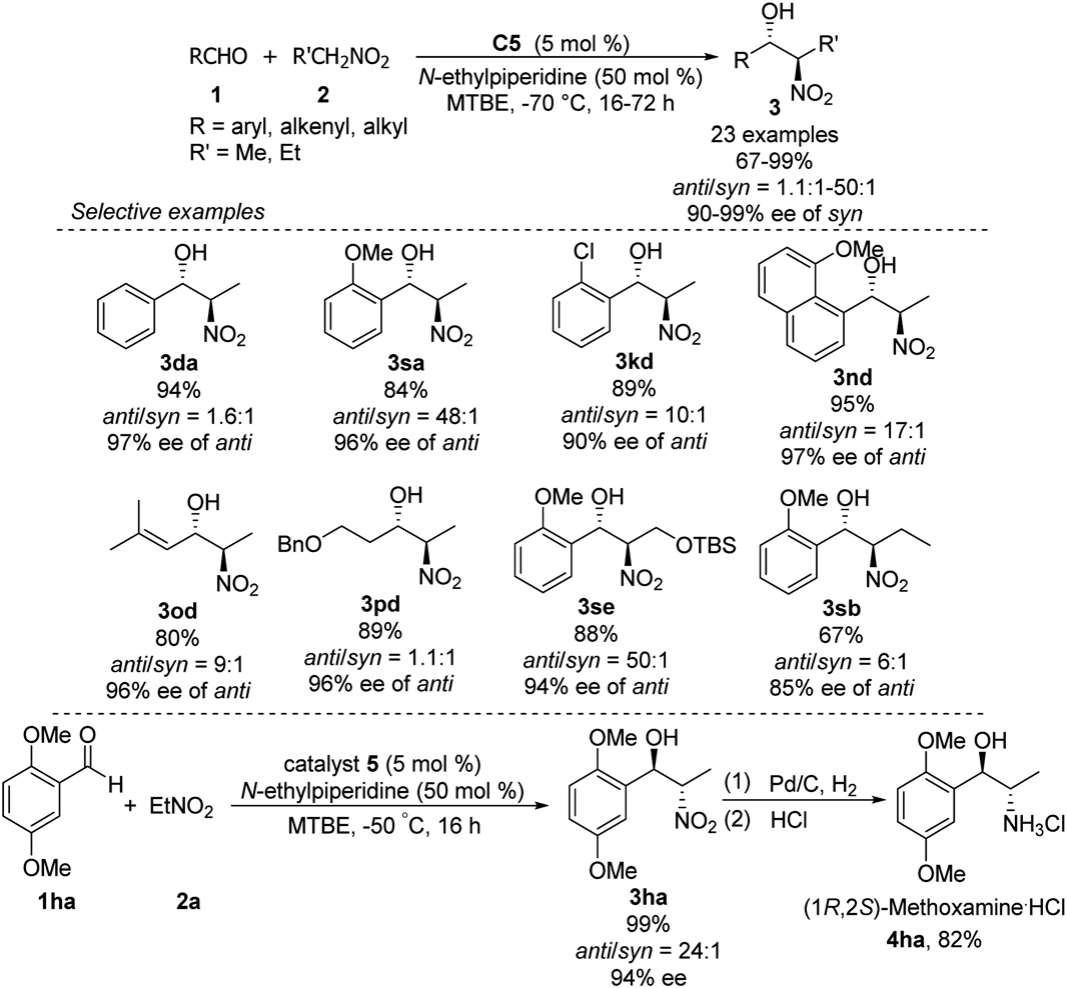
Hong's Synthesis of (1*R*,2*S*)-methoxamine hydrochloride.

High *anti*-selectivity was obtained using methyl *tert*-butyl ether as solvent and *N*-ethylpiperidine as base, but this selectivity fell to an *anti*/*syn* ratio around 2 : 1 when the substrate was benzaldehyde without an *ortho* substitution (Scheme 29). The catalyst 5-promoted Henry reaction was applied to the synthesis of (1*R*,2*S*)-methoxamine hydrochloride 4ha, an α1-adrenergic receptor agonist.

## 5. Organocatalytic asymmetric diastereoselective Henry reactions

### 5.1. Guanidine derived organocatalysts

**Scheme 30:**
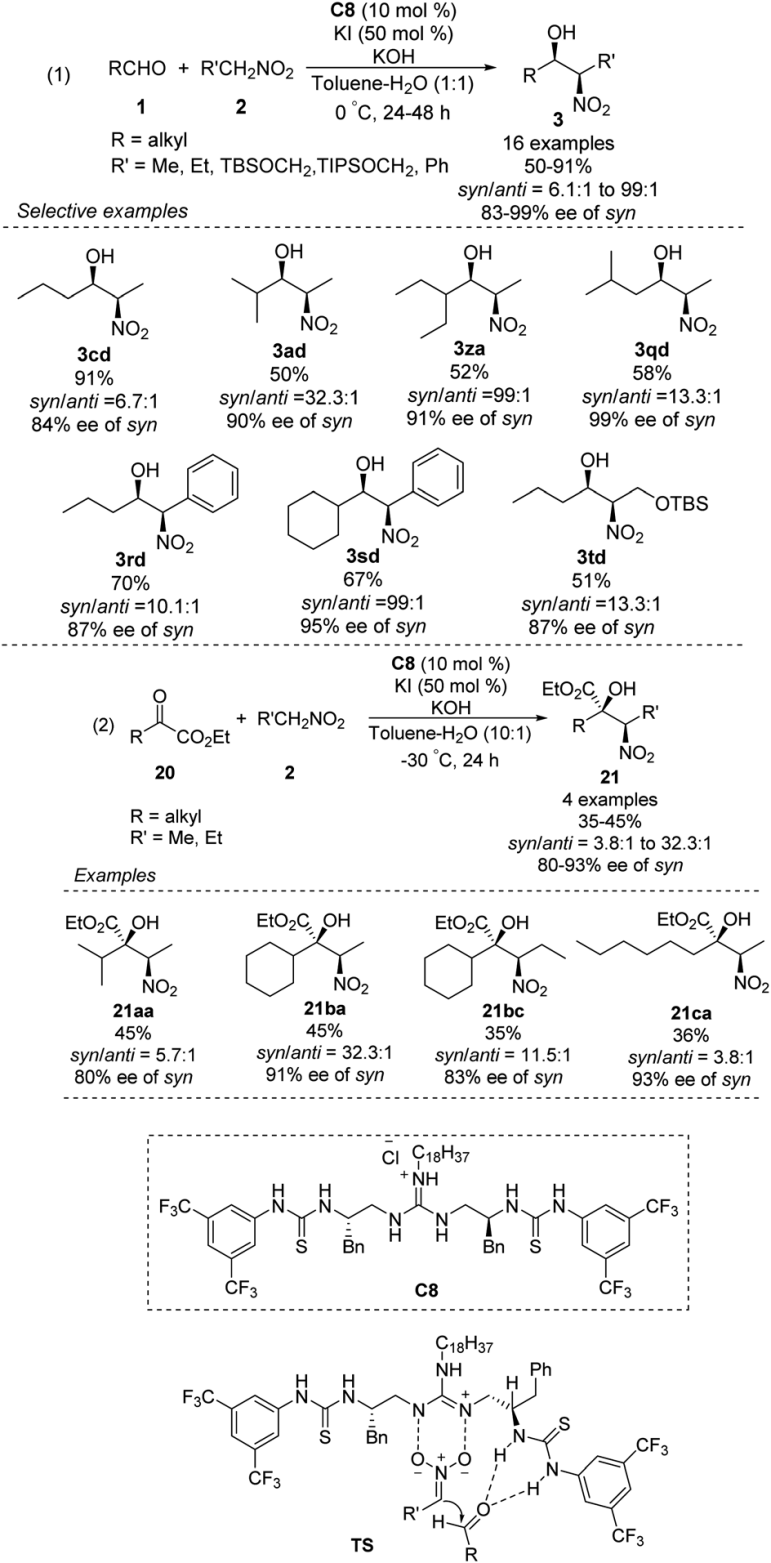
Guanidine-thiourea C8 catalyzed Henry reaction.

The Nagasawa group achieved the first organocatalytic asymmetric diastereoselective Henry reaction in 2006.^55^ The guanidine–thiourea bifunctional organocatalyst C8 catalyzed reaction between various aliphatic aldehydes and nitroalkanes (Scheme 30). Yields were moderate to good, and *syn*-selective products were obtained with high enantioselectivity. The inorganic salt KI proved crucial for inhibiting the *retro*-nitroaldol reaction and for improving enantioselectivity. The same organocatalyst C8 was extended to the reaction of nitroalkanes with different α-keto esters, giving products in moderate yields with moderate enantioselectivities and high *syn* selectivity.^56^ Transition state of the Henry reaction catalyzed by C8 was based on the chemoselective dual activation concept.^57^

**Scheme 31:**
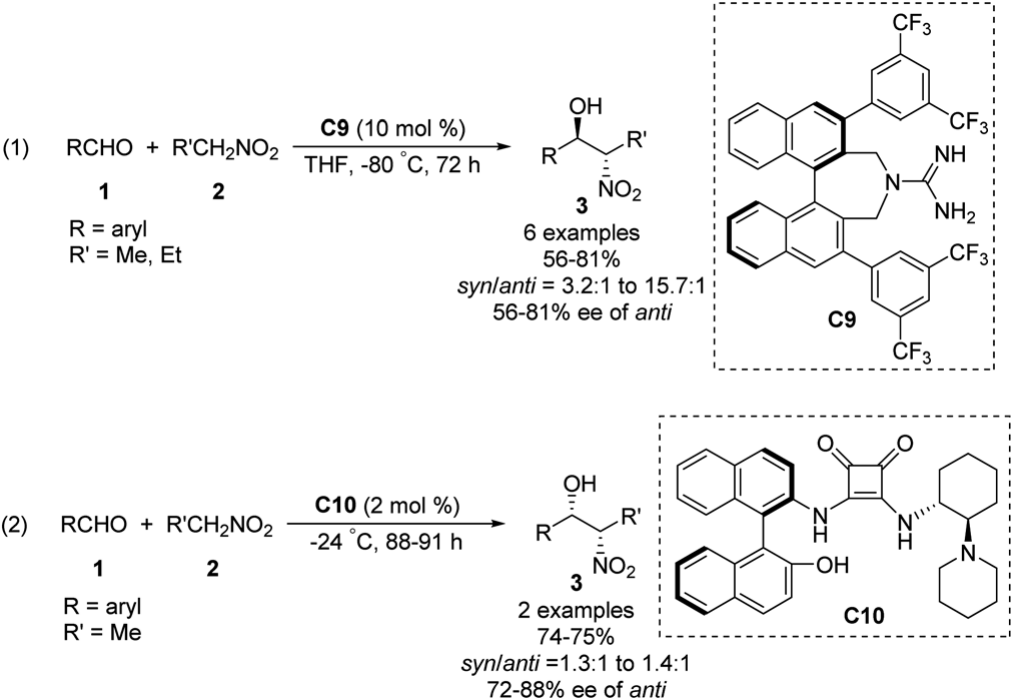
Axially chiral guanidine catalysed Henry reactions.

Then, the Terada group^58^ and the Herrera group^59^ independently reported the diastereo- and enantioselective Henry reactions of nitroalkanes with aldehydes using axially chiral guanidine C9 and C10 as the catalyst. However, optically active products were obtained in moderate yield and poor enantio- and diastereoselectivities (Scheme 31).

### 5.2. Tetraaminophosphonium salt derived organocatalysts

**Scheme 32:**
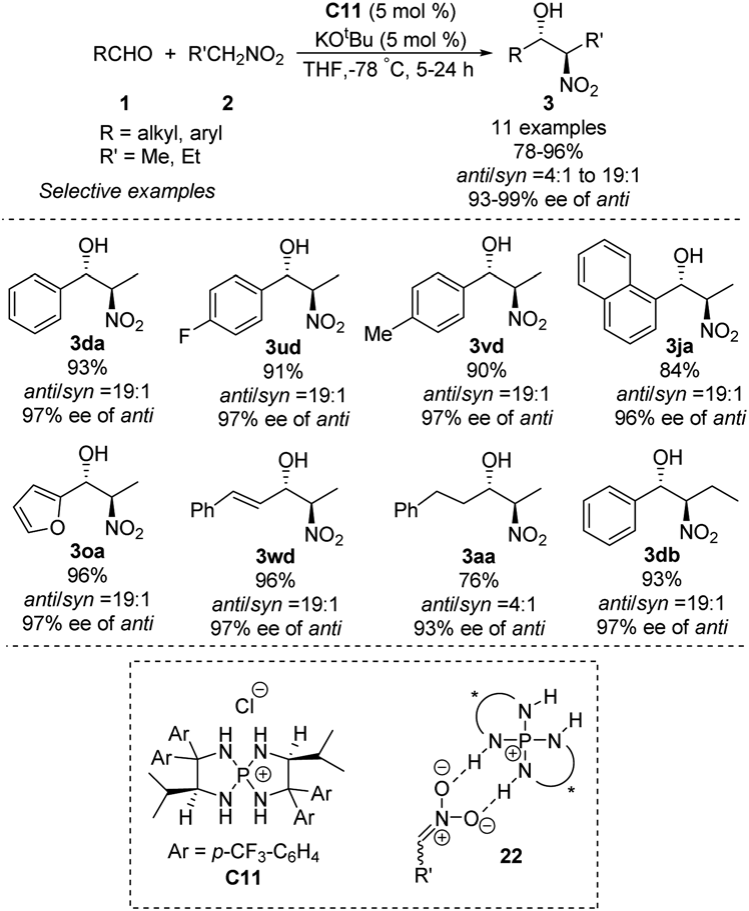
Tetra-aminophosphonium salt-catalyzed Henry reactions of aldehydes.

Asymmetric phosphine catalysis has emerged as a remarkable and powerful strategy for constructing chiral molecules,^60^ but few such reactions involve quaternary phosphonium salts. In 2007, Ooi used chiral *P*-spirocyclic tetra-aminophosphonium salts to promote asymmetric diastereoselective Henry reactions.^61^ In reactions of aromatic aldehydes with nitroethane or nitropropane, the catalysts provided enantioselectivity up to 99% ee and *anti*/*syn* ratios up to 19 : 1 (Scheme 32). Using aliphatic aldehydes resulted in moderate yields, enantioselectivities and diastereoselectivities. The reaction has been proposed to proceed *via* an ion pair complex 22 that forms when nitronate anions hydrogen-bond to the secondary amino group of catalyst C11.

**Scheme 33:**
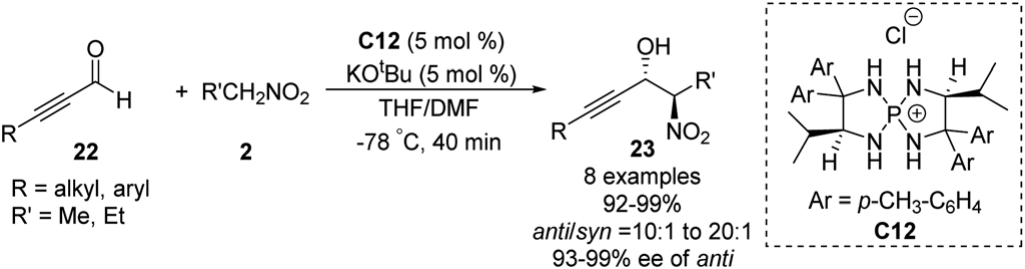
Catalytic asymmetric direct Henry reaction of ynals.

**Scheme 34:**
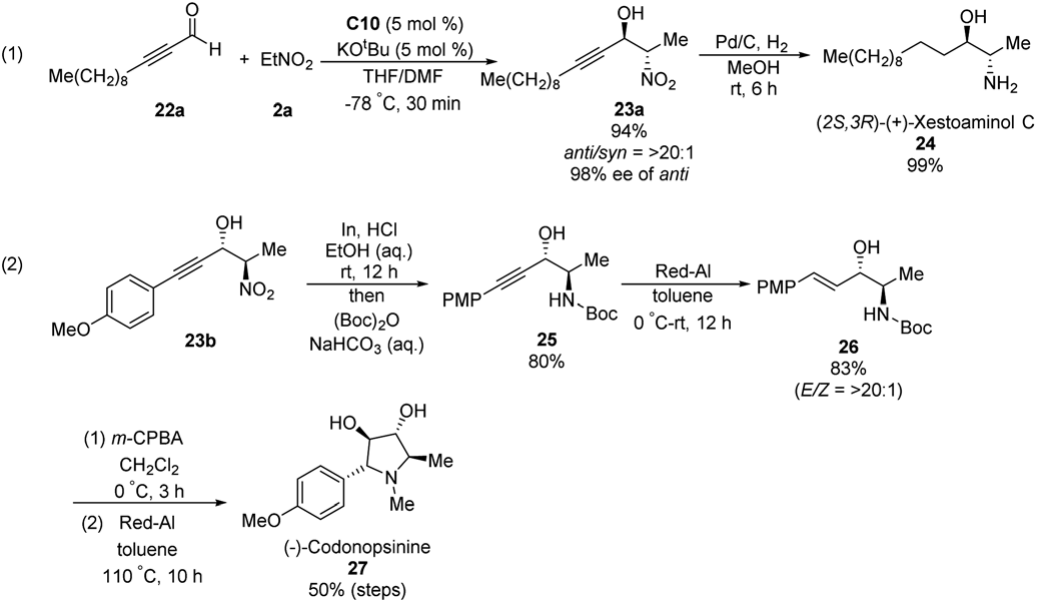
Short syntheses of (*2S*,*3R*)-(+)-xestoaminol C and (−)-codonopsinines.

Ooi extended the scope of this approach to ynals, a relatively unexplored substrate in asymmetric Henry chemistry (Scheme 33).^62^ Adding *N*,*N*-dimethylformamide as a co-solvent suppressed decomposition of the aminophosphonium alkoxide intermediate. Aromatic and aliphatic ynals were suitable substrates, affording the corresponding propargylic alcohols in excellent yields, enantioselectivities, and diastereoselectivities. Through this approach, (2*S*,3*R*)-(+)-xestoaminol C (24) and (−)-codonopsinine 27 were synthesized concisely (Scheme 34).

### 5.3. Cyclodextrins derived organocatalysts

**Scheme 35:**
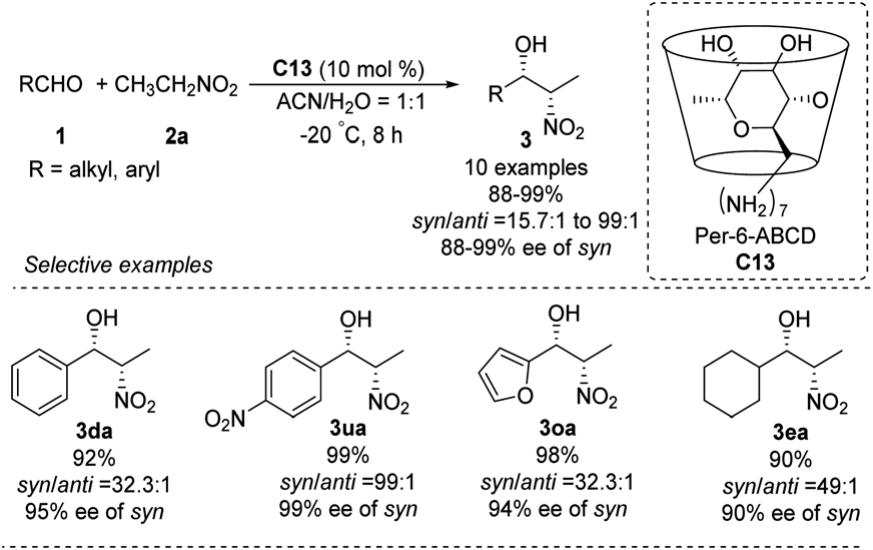
Per-6-amino-β-cyclodextrin C13 catalyzed Henry reactions.

In 2010, the Pitchumani group used per-6-amino-β-cyclodextrin C13 to catalyze the highly *syn*-selective Henry reaction of nitroethane with different aldehydes (Scheme 35).^63^*Syn*-products were formed in good yields and enantioselectivities. The catalyst was easily recovered by simple filtration, and reused without loss of activity. So far, however, catalyst C13 has not been applied to other nitroalkanes.

### 5.4. Cinchona alkalloid derived organocatalysts

**Scheme 36:**
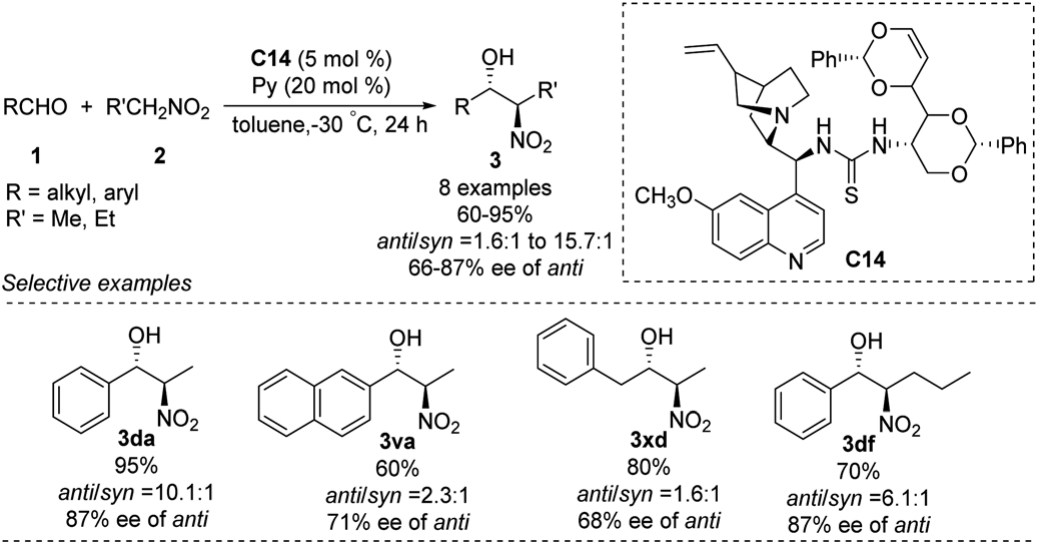
Cinchona alkalloid catalyst C14 catalyzed Henry reactions.

The He group reported the use of a new family of cinchona alkaloid-thiourea catalysts in *anti*-selective asymmetric Henry reactions.^64^ Catalyst C14 led to isomers in yields up to 95%, an *anti*/*syn* ratio of 91 : 9 and enantioselectivity of 87% ee (Scheme 36). This catalyst worked even in water: it afforded products in up to 93% yield, an *anti*/*syn* ratio of 94 : 6 and enantioselectivity of 88% ee in toluene/water (7 : 3). In contrast, reacting aliphatic aldehydes with nitroethane gave an *anti*/*syn* ratio of only 1.6 and enantioselectivity of only 68% ee.

## 6. Conclusions

Although lagging behind the extensive literature on asymmetric Henry reactions to form chiral β-nitro alcohols, the development of one-pot catalytic diastereoselective nitromethane-free Henry reactions to generate chiral β-nitro alcohol scaffolds with four adjacent stereogenic centers has been impressive. Here we have reviewed several reactions using metal- or organo-catalytic systems to react unfunctionalized higher nitroalkanes such as nitroethane or nitropropane with carbonyl compounds in a highly enantio- and diasteroselective manner. Despite these advances, at least three substantial barriers remain. First, direct catalytic asymmetric Henry reactions in which the less reactive ketone carbonyl can serve as electrophile remain rare. Second, stereoselectivity is often poor when the substrate is a substituted nitroalkane such as bromonitromethane, 1-bromo-2-nitroethane or 2-nitroethanol. Third, chiral ligands are usually expensive to purchase or difficult to synthesize. As more efficient catalytic systems are developed, we believe that these problems will be solved, and the range of applications for these Henry reactions will expand.

## Conflicts of interest

The authors declare no conflict of interest.

## Supplementary Material
